# Towards responsible digital health implementation: A mixed-methods exploratory study developing a tool to assess workforce experience

**DOI:** 10.1177/20552076261450419

**Published:** 2026-07-06

**Authors:** Bazuin Tom, Michiel Sebastiaan Oerbekke, Pauline Heus, Mike Petrus Theodora Kusters, Dave Anton Dongelmans, Annelies Visser, Arie Franx, Johann Klaus Götz Wietasch, Lotty Hooft, Maarten Jurriaan van der Laan

**Affiliations:** 1 Department of Surgery, University of Groningen, 10173 University Medical Centre Groningen, Groningen, The Netherlands; 2 Julius Centre for Health Sciences and Primary Care, University Medical Centre Utrecht, Utrecht University, Utrecht, The Netherlands; 3 Cochrane Netherlands, University Medical Centre Utrecht, Utrecht University, Utrecht, The Netherlands; 4 Department of Intensive Care Medicine, 26066 Amsterdam UMC Location University of Amsterdam, Amsterdam, The Netherlands; 5 Department of Surgery, Amsterdam University Medical Centre, University of Amsterdam, Amsterdam, The Netherlands; 6 Department of Obstetrics and Gynaecology, Erasmus MC, University Medical Centre Rotterdam, Rotterdam, The Netherlands; 7 Department of Anaesthesiology, University of Groningen, 10173 University Medical Centre Groningen, Groningen, The Netherlands

**Keywords:** digital health < general, healthcare professional, tool, impact, wellbeing, artificial intelligence < general, HCP

## Abstract

**Background:**

Digital health technologies (DHTs) are increasingly implemented to improve efficiency, quality, and patient experience. However, conditions that could influence the impact on well-being of healthcare professionals (HCPs) are rarely assessed.

**Objective:**

To develop a tool to support policymakers, hospital administrators, and implementation teams in considering workforce experience and well-being implications of DHTs on HCPs.

**Methods:**

A mixed-methods study was conducted in three Dutch university medical centres. First, we re-analysed a scoping review on DHT-implementation, applying criteria focusing on HCP-related determinants and thematically analysing these within a modified Work-System model and Quintuple Aim framework. Second, we conducted a prioritisation survey using the Reweighted Priority-Setting tool to rank themes related to *Care-team well-being*, one of the Quintuple Aim domains. Third, we explored the highest-ranked themes in focus-groups and interviews with HCPs and stakeholders. Finally, we synthesised findings into a tool.

**Results:**

From 73 eligible studies we extracted determinants, generating themes and subthemes, of which 44 mapped to *Care-team well-being*, and were used as survey items. Twelve respondents (eight HCPs and four policy-staff) prioritised these. Top-ranked themes included perceived benefits, acceptance, involvement in development and implementation, ‘champions’, training and skills, communication, and role clarity. Analysis of two focus-groups and three interviews yielded six domains: acceptance, champions, process and development, role of the professional, communication, and training. These informed a 14-item checklist for use during DHT-implementation.

**Conclusion:**

We developed a reflective checklist to support structured consideration of conditions shaping workforce experience in DHT-implementation. The tool offers a starting point for embedding workforce conditions in DHT-governance.

## Introduction

Digital health technologies (DHTs) are increasingly embedded in healthcare systems worldwide.^1,2^ These technologies are promoted as means to improve care quality, streamline workflows, enable personalised care, reduce the workload of healthcare professionals (HCPs), and the pressure on the health system.^1,3–5^ However, despite these promises, the impact of DHTs on HCPs, remains insufficiently assessed in most implementation processes. Evidence shows that DHTs may unintentionally increase workload, generate role ambiguity, and contribute to technostress, while potential benefits for HCPs are often uncertain or delayed.^3,6–10^ Although HCP well-being is a core component of the Quintuple Aim,^11,12^ their perspectives are rarely incorporated in a consistent way during DHT development and implementation.^12^ As a result, effects on workload, professional roles, and day-to-day practice remain largely invisible, even though these factors critically influence adoption, quality of care, and long-term sustainability.^10,13–15^

Multiple reviews have documented barriers and facilitators influencing HCP adoption of DHT and highlighting recurring issues such as workflow disruption, usability, training requirements, and competency gaps.^10,16–18^ These studies describe determinants across settings and professional groups, including implementation barriers and facilitators, and outline competencies required for effective use. However, these insights are typically reported as conceptual determinants or competency domains and provide limited guidance on how implementation teams can translate them into concrete, routine prompts for governance decisions and implementation planning.

Insufficient attention to HCPs’ perspectives risks undermining not only staff well-being but also the intended patient benefits of DHTs, as professionals are then less able or willing to integrate new tools effectively into their workflow.^19–21^ Understanding HCPs’ experiences with DHTs requires a sociotechnical lens on how technologies interact with people, teams, norms, values, workflows, and power structures within healthcare work systems.^22^ Several implementation frameworks consider professional perspectives to some extent, including the Technology Acceptance Model, Measurement Instrument for Determinants of Innovation, and the Consolidated Framework for Implementation Research.^23–25^ These frameworks identify relevant determinants, but offer limited guidance for evaluating, documenting, and acting on the impact of DHT implementation on HCPs. These frameworks fail in translating conceptual determinants into concrete prompts for daily practice, and a HCP-specific framework is currently missing.

Although determinants of DHT implementation and relevant competency domains are increasingly well described, implementation teams still lack concise and pragmatic tools to systematically reflect on workforce experience and on implementation conditions that may safeguard or undermine HCP well-being during DHT implementation.^17^ This gap may hinder informed decision-making and may contribute to implementation failure or increased workforce strain. To address this gap, we aimed to develop an evidence-informed and pragmatic tool that supports policymakers and implementation teams in assessing how DHTs affect HCPs, and in integrating these considerations into implementation planning and governance.

## Methods

### Study design and setting

**Figure 1.**
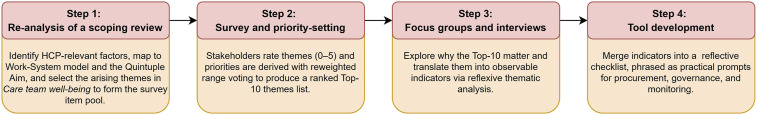
The figure above illustrates how the different phases of this study follow each other and how these eventually led to the creation of the tool.

We conducted a multi-step, sequential mixed-methods exploratory study as part of a collaboration of three Dutch university medical centres. We performed a re-analysis of a scoping review within this collaboration (Step 1) (Kusters et al., submitted), a priority-setting assessment (Step 2), focus-groups and interviews (Step 3), and tool development (Step 4). Results from Step 1 informed the survey in Step 2. Prioritised outcomes from Step 2 guided the topics for the focus-groups and interviews in Step 3. Results from Step 3 were used to develop the tool in Step 4. A study flow diagram is provided in Figure 1.

#### Step 1: Re-analysis of a scoping review

##### Step 1.1: Methods of the original scoping review

This study builds on a scoping review conducted within our collaboration (Kusters et al., submitted). The review searched MEDLINE (via Ovid) and Embase (via https://Embase.com) for peer-reviewed studies published in Dutch or English between 2014 and March 2024. An information specialist developed the search strategy combining terms for digital health technology, hospital setting, and implementation determinants (full strategies in Supplementary material, Text S1). As this was a review of published studies, ethical approval was not applicable and the protocol was not registered. Titles/abstracts and full texts were screened independently by two reviewers. Discrepancies were resolved through discussion and, if necessary, consultation with a third reviewer. The review included quantitative, qualitative, mixed-methods, proof-of-concept studies, and reviews reporting implementation determinants (e.g., barriers, facilitators, indicators) related to the implementation of patient-oriented DHTs in hospital settings, without geographical restrictions. Technologies were considered patient-oriented when they provided services directly to patients (e.g., self-monitoring, telemonitoring). Studies focusing solely on technologies aimed at healthcare professionals (e.g., electronic health records) were excluded. Determinants were extracted using a standardised form. Data extraction was performed by one reviewer and checked by a second. Implementation determinants were classified using the Tailored Implementation for Chronic Diseases (TICD) checklist.^26^

##### Step 1.2: Re-analysis of the original scoping review

For the present study, we re-analysed the scoping-review dataset using stricter criteria focused on workforce considerations. We included only studies that explicitly reported determinants, barriers, or facilitators related to HCPs’ work, roles, workload, competencies/training, acceptance, or other conditions affecting workforce experience in DHT implementation. We excluded studies that focused solely on patient uptake or patient outcomes without explicit HCP-related determinants. Screening decisions for the re-analysis were made by two reviewers. Discrepancies were resolved through discussion and, if necessary, consultation with a third reviewer. From the eligible studies, TB extracted HCP-related determinants (barriers and facilitators), which were verified by MK. Determinants were then analysed using Braun and Clarke’s reflexive thematic analysis.^27^ TB conducted coding and theme development, while MK reviewed and refined the codes and themes. Disagreements were resolved through discussion until consensus was reached. To explore how determinants interact within complex work systems, the themes were subsequently mapped onto a modified Work-System Model capturing interactions between technology, tasks, people, and organisational context.^28^ This mapping was performed by TB and checked by MK. A detailed description of the analytic process and modifications to the Work-System Model is provided in the Supplementary Material (Text S2).

#### Step 2: Survey and priority-setting

The themes mapped on the modified Work-System Model were classified following the five categories of the Quintuple Aim.^12,29^ All themes assigned to *Care-team well-being* were selected and converted to survey items. The survey, designed to support prioritisation rather than statistical inference, used a six-point scale (0 = no priority; 5 = high priority) to elicit priority scores from participants. We used purposive and snowball sampling via institutional mailing lists of the centres where the authors work, and professional networks to recruit survey participants. This was chosen in order to recruit an information-rich group with direct experience in DHT implementation. Participants were eligible if they were HCPs (e.g., physicians, nurses) or hospital employees (e.g., IT specialists, project managers) with current or recent involvement in DHT-implementation within hospital settings. The survey was open throughout November 2024 and administered via the Research Electronic Data Capture (REDCap) web application.^30,31^ The survey was offered in Dutch, and later translated to English, for this publication (supplementary materials, Text S3). The survey aimed to obtain scores for the priority-setting of items, which would inform further qualitative exploration and tool development. We included submissions with 100% item completion. After obtaining the scores, we used a reweighted range voting approach using the Reweighted Priority-Setting (REPS) tool to obtain a ranked list of themes based on priority scores.^32^ Participants score each theme on a fixed scale and these scores are the input for the REPS tool. The tool uses reweighted range voting to reweigh each participant’s influence after assigning a rank to a theme. The sum of the individual participant’s scores on previous ranked themes result in a decline of their weight for assigning a rank to the next theme. The higher their previous scores on ranked themes, the lower their weight. This makes REPS suitable for situations where the goal is to reflect group priorities,^33^ even in small, information-rich samples. Note that it is not a consensus method. We used the unmodified range voting method in the tool. Any ties during ranking with the tool were resolved by selecting the theme with the highest unweighted sum score first. The REPS-tool’s top-10 ranked themes were selected for qualitative exploration. It functioned as a pragmatic funnel, providing focus from the broad evidence base to specific topics of interest for in-depth qualitative exploration and checklist development. As a sensitivity check, we also performed an unweighted sum score ranking of all themes.

#### Step 3: Focus-groups and interviews

Two focus-groups of approximately 120 minutes each were organised through online video calls in December 2024 to facilitate participation across sites. Participant recruitment was done similarly to that for the survey. We used purposive sampling to capture variation in profession, and hospital type among stakeholders. Consistent with our constructivist perspective and qualitative study aim, we did not seek a statistically representative sample. Instead, we aimed to capture a broad range of perspectives through purposive sampling across professional roles and organisations. Topic guides were developed to ensure a semi-structured approach for the focus-groups (Supplementary materials, Text S4). In the focus-groups we aimed to explore why each prioritised theme matters to HCPs in terms of well-being, and to explore observable indicators that a priority theme is adequately addressed during implementation. Focus-groups were moderated by TB, with MO observing. Recordings were collected online via Microsoft Teams, where initial computer-generated verbatim transcripts were de-identified and further hand corrected by one researcher (TB) using the recordings. The same topic guide was used for three one-on-one online semi-structured interviews with purposefully sampled participants. With these interviews, we aimed to reach additional depth to the topic. One researcher (TB) conducted the interviews. Both the focus-groups and the interviews were conducted in Dutch. We used Braun and Clarke’s reflexive thematic analysis to analyse the data.^27^ Coding and theme development were iterative. TB conducted initial open coding in Dutch, followed by clustering codes into candidate themes. MO reviewed the developing themes. Discrepancies and interpretive decisions were discussed between TB and MO until consensus on the thematic structure was reached. To enhance reflexivity, themes were also discussed with co-authors, who were medical specialists, implementation experts, and epidemiologists. Throughout the analysis, we used analytic memos to document coding decisions and theme development Themes were then translated to English. Based on the richness and relevance of the collected data, we considered the dataset to provide sufficient information to support the development of an implementation oriented checklist. Ultimately, for this publication, themes were translated to English.

#### Step 4: Tool development

Results from the prioritised themes in Step 2 and the RTA in Step 3 were used to generate the tool’s items. These items were themes re-phrased as guiding questions or prompts to support structured reflection on whether and how each aspect was addressed during DHT-implementation. Qualitative analyses in Step 3 provided additional context for each item of the tool. Items were merged when survey themes and qualitative insights consistently reflected the same underlying mechanism. Only themes supported by both prioritisation data and qualitative findings were retained as separate tool items, ensuring that the final tool balanced comprehensiveness with feasibility. Draft items were iteratively refined for clarity and feasibility using feedback from co-authors.

### Ethics and reporting

Review by a Medical Research Ethics Committee was not required since the study is not under the scope of the Dutch Medical Research involving Human Subjects Act.^34^ Participants provided informed e-consent for survey participation in REDCap,^30,31^ and provided written informed consent for participation in interviews or focus groups by mail. Reporting followed the Good Reporting of A Mixed Methods Study (GRAMMS) checklist. For the scoping review part, the Preferred Reporting Items for Systematic reviews and Meta-Analyses extension for Scoping Reviews (PRISMA-ScR) checklist was used. The qualitative part was reported in accordance with the Consolidated criteria for reporting qualitative studies (COREQ). (Supplementary material, Text S5-S7).

## Results

### Results from the scoping review re-analysis step

The initial scoping review retrieved 2,687 records. The included studies in the original review, can be found in the supplementary material (Table S1 and Text S8). Seventy-three studies met the eligibility criteria of our re-analysis. A combined selection flow diagram, and reasons for exclusion, can be found in the supplementary material (Figure S1, Table S2). From the included studies, we extracted 501 determinants relating to HCPs, which were coded into 647 labels and mapped onto the Work-System Model. Because many determinants reported by HCPs reflected patient-mediated factors, we added an explicit patient domain and described interactions between domains. The thematic synthesis yielded 52 themes, and 43 subthemes, which were mapped to the Quintuple Aim domains. Forty-four mapped to the *Care-team well-being* domain and served as input for the prioritisation survey. All themes and mapping can be found in the supplementary material (Table S3a and S3b, Figure S2).

### Results from the survey and priority-setting step

Twelve respondents, HCPs (n = 8, 67%) and managers or policy staff (n = 4, 33%), completed the full prioritisation survey. Participant characteristics can be found in Table 1. Most respondents had more than six years of experience in healthcare (n = 8, 67%) and reported daily use of DHTs (n = 9, 75%). We ranked themes using the REPS procedure, selecting the top-10 for qualitative exploration (Table 2). A full overview of ranking can be found in the supplementary material (Table S4). Participants were purposively selected for experience with DHT-implementation. Ranking based on the unweighted sum score shows that the same 10 items remain in the top-10, although position 10 is then tied with four other items (Supplementary material, Table S5).

**Table 1.** Characteristics of survey participants.

Item	Response	n (%)
Complete responses	-	12 (100%)
Current role	Healthcare professional	8 (67%)
	Manager	3 (25%)
	Policy staff	1 (8%)
Years in healthcare	1–5 years	4 (33%)
	6–10 years	3 (25%)
	11–20 years	3 (25%)
	≥20 years	2 (17%)
Frequency of DHT use	Daily	9 (75%)
	Weekly	2 (17%)
	Monthly	1 (8%)
Type of technology*	Apps	6 (50%)
	Telemonitoring	5 (42%)
	Patient portal	3 (25%)

*Type of technology exceeds 100% as respondents could select multiple options. *DHT: digital health technology*.

**Table 2.** Top-ranked themes influencing DHT impact on healthcare professionals.

Rank	Theme
1	Perceived benefits of DHT use by healthcare professionals
2	Acceptance and use of DHTs by healthcare professionals
3	Involvement of healthcare professionals in the implementation process
4	Presence of leaders and champions
5	Involvement of healthcare professionals in the development process
6	Involvement of appropriate stakeholders
7	Training, education, and support
8	Training, education, and support: skills development
9	Communication between the organisation and healthcare professionals
10	Clarity regarding the role of healthcare professionals in the process

*DHT: digital health technology.*

### Results from the focus-group and interview step

We conducted two online focus-groups (n=3; n=4) and three individual interviews in December 2024. Participant characteristics are presented in Table 3. Focus-group participants were active at policy-/project-level around DHT, interviews were with HCPs, all from different organisations. After the first focus-group, the topic guide was refined, and overlapping themes were merged, yielding six categories that structured subsequent interviews and the second focus-group: acceptance, champions, process & development, role of the professional, communication, and training.

**Table 3.** Role or clinical profession of focus-group and interview participants.

Method	Number	Participant	Role/Profession
Focus-group	Group 1	P1	Cardiologist
		P2	Project lead
		P3	Nursing supervisor
	Group 2	P4	Chief Nursing Information Officer
		P5	Product owner eHealth
		P6	Health Innovation Advisor
		P7	Health Innovation Policy Officer
Semi-structured interview	Interview 1	P8	Nurse
	Interview 2	P9	Medical doctor
	Interview 3	P10	Nurse

#### Acceptance

Acceptance of DHTs was described as dependent on the perceived balance between short-term effort and long-term benefit. Participants acknowledged that initial implementation requires additional time for training and patient instruction, with efficiency gains becoming apparent later. Where these expected future benefits were considered credible, staff were more willing to tolerate the initial investment. As one participant noted:

‘*’By actually showing them (NB. HCPs) what it will bring them (…) So that way (…) it costs at the beginning, it requires an investment of time, but the return is greater*.’’ (P9)

Participants noted that acceptance differed across professional groups, often related to variation in experience and familiarity with DHT rather than age. While broad inclusion of HCPs during implementation was viewed as important, several argued that once a DHT demonstrates value, organisations should establish norms to ensure adoption, even among reluctant staff.

#### Champions

Champions, trusted and motivated colleagues within the same professional group, were seen as critical to align other team members. Unlike general enthusiasts, champions actively mobilise others, translate innovations into practice, and sustain momentum. Their influence was strongest when they operated as peers within their team. Misalignment between champion and affected team often undermined uptake. Participants emphasised that multiple champions are often needed to bridge teams and ensure broad support.

#### Process & development

Early involvement of HCPs in the development and implementation of DHTs was repeatedly identified as essential to ensure usability and prevent frustration. Subsequently, participants described multidisciplinary working groups, including representatives from each affected role as effective in surfacing problems early. Not only was this related to HCPs, but the inclusion of IT specialists and legal advisors was also considered important, to avoid pushing decisions onto clinicians that lie outside their expertise. Furthermore, transparent communication about realistic timelines was another recurring theme: professionals cautioned that the process often spans years rather than months, and that unclear expectations could lead to demotivation or resistance. And lastly, hospitals with a centralised DHT-desk or coordination team were regarded as particularly effective in supporting implementation, connecting relevant stakeholders, and avoiding duplication of efforts. In contrast, its absence was perceived as a shortfall to unburden the HCP.

#### Role of the professional

Clarity about roles and responsibilities was viewed as a prerequisite for sustained engagement with DHTs. Ambiguity around who is responsible for particular tasks or decisions was said to cause inefficiency, frustration, and reduced motivation for the HCP. Conversely, clear implementation plans in which responsibilities are explicitly defined were perceived as facilitating smooth adoption, positively reflecting on the HCP. Participants stressed that DHT should be designed to make work easier rather than creating additional (administrative) burden.

#### Communication

Participants highlighted the role of departmental leads, such as head nurses, as crucial intermediaries who translate organisational information into practically relevant messages for the frontline staff. Timely updates were described as motivating, whereas lack of communication was associated with uncertainty and resistance. As one professional noted:

“*That’s already a first step in creating motivation: giving people a little stimulus by showing: we’re working on this. (…) Then you already have some awareness of what’s going on and what’s coming. (…) If you’re informed at an earlier stage, (…) that really contributes to motivation.*” (P8).

#### Training

Participants stressed that training should be structurally embedded in the organisation, for example, offered during working hours and incorporated into onboarding programmes, to ensure that all professionals are adequately supported. Crucially, participants distinguished between two types of competences: technical skills, such as navigating an application, which can be taught relatively easily, and clinical competences, such as deciding how to act on remotely monitored patient data, which require more context-specific discussion and role clarity. Without support across these domains, HCPs will likely fall back on old routines or avoid the technology altogether.

#### Cross-cutting observation: Difficulty articulating HCP-focused impacts

Across themes, participants found it challenging to articulate how DHTs affected their well-being, even when directly prompted to reflect on it. Discussions often shifted toward patient benefits or workflow efficiency rather than personal or collective impacts on staff. This difficulty was unexpected, as one of the main aims of the focus-groups and interviews was to explore well-being more explicitly. The finding itself is therefore meaningful: it illustrates how the well-being of HCPs remains a more implicit, under-discussed topic in digital transformation processes. Participants reflected on this imbalance, noting that data and evaluation mechanisms predominantly focus on patients rather than professionals. The cardiologist in our focus-group observed:

“*See, we measure a lot for patients. It’s a bit funny right? Because (…) we ask healthcare professionals far less*.” (P1).

This underscores both the need and the challenge of systematically integrating HCP well-being into DHT-implementation. It also helps explain why the resulting tool emphasises work-related factors influencing staff experience, rather than well-being outcomes.

### Results from the tool development step

Based on the six overarching themes identified in the qualitative analysis we developed a 14-item reflective checklist. The checklist translates themes into actionable prompts that support implementation teams in evaluating how DHT may affect HCPs and whether essential conditions for successful uptake are in place. Table 4 provides an overview of the checklist items, accompanied by a column for documenting how each aspect is addressed during the implementation process.

**Table 4.** Checklist for evaluating the impact of DHT on healthcare professionals (6 themes, 14 items).

Question	Item	How is this item taken into account?
*New DHTs should not burden healthcare professionals unnecessarily. Time investments in DHT must take place during working hours and ultimately result in time savings.*	Acceptance, item 1: Time investment	​
**Question**: Is the DHT designed to save the healthcare professional time, both in the short and long term? Explain. If there is an initial time investment, describe how this pays off and how it converts into time savings over time.		
*Generational differences can influence the acceptance of DHT. Especially older healthcare professionals often experience more resistance and find the changes more difficult, while younger generations consider technology self-evident.*	Acceptance, item 2: Generational differences	​
**Question**: How are generational differences among healthcare professionals taken into account in the implementation of the DHT? Explain.		
*Voluntariness in the use of proven effective DHT can hinder efficiency and quality of care. Sometimes it is necessary to act in a norm-setting way to promote broad acceptance.*	Acceptance, item 3: Norm-setting	​
**Question**: How does the organisation ensure broadly adopted DHT? Explain, and describe when norm-setting action is taken.		
*Actively gathering input and having periodic evaluation moments for DHT are essential to identify where the healthcare professional encounters obstacles or what is appreciated, and thereby promote acceptance of technology.*	Acceptance, item 4: Evaluation	​
**Question**: Are structured evaluation moments built in during and after implementation to collect and process feedback from healthcare professionals? Explain.		
*The presence of ‘champions’, or ‘early leaders’, in the implementation of DHT leads to greater acceptance among care providers and successful implementation. They are able to motivate a large group of people to use the technology. To get this group on board, it is also important that the champion is an equivalent healthcare professional, in other words, a peer. In addition, it is important to keep this group motivated.*	Champions, item 5: Presence of champions	​
**Question**: Have champions been identified who can enthuse and motivate others during the implementation process? Describe how their presence is ensured, why the right champion has been identified for the target group, and how this group remains enthusiastic and engaged.		
*Involving healthcare professionals in the development of DHT increases usability and prevents frustration or incorrect use. With DHT where there are tasks for the healthcare professional, it is important to develop the technology with their input as well, to ensure that the wishes and needs of that professional group are incorporated at an early stage.*	Process and development, item 6: Involving the healthcare professional in development	​
**Question**: Are healthcare professionals involved from the beginning in the development of DHT? Explain how that was done.		
*A working group with representation from all involved professional groups can ensure a smooth implementation of DHT by identifying and solving concerns and bottlenecks at an early stage. The representatives from the professional groups can also act as a contact person between the other healthcare professionals and the team working on the technology. In the same vein, it is also important for the healthcare professional that the right other stakeholders are involved, for example, IT staff or people knowledgeable about legal aspects. This prevents healthcare professionals from being made responsible for matters about which they have insufficient knowledge.*	Process and development, item 7: Setting up a working group	​
**Question**: Has a working group been set up with representatives from all involved professional groups, including healthcare professionals, and other stakeholders? Explain how this team is composed and how the representatives from the professional groups inform their constituencies.		
*Clear communication about the duration and complexity of implementation prevents unrealistic expectations and loss of motivation among healthcare professionals. This can help to prevent promising innovations from failing at an early stage.*	Process and development, item 8: Duration and complexity of implementation	​
**Question**: From the start, is transparent communication provided about time and complexity of implementation of DHT? Explain how this is done.		
*Arranging DHT centrally within a hospital, for example, through a DHT helpdesk, facilitates implementation and provides healthcare professionals with the necessary support. Creating a central point of contact for DHT ensures better coordination of innovations, rapid matching of the right personnel, and prevents unnecessary duplication of efforts.*	Process and development, item 9: Central arrangement	​
**Question**: Is there a central helpdesk or coordinator for DHT within the hospital? Explain how this is organised and how such a helpdesk has been used for the current innovation.		
*Clear division of roles and responsibilities in the implementation of DHT is essential for successful use by healthcare professionals. When healthcare professionals know what is expected of them and which specific tasks they must perform, they remain motivated and contribute to the process. It is important that an implementation plan explicitly elaborates the roles in order to prevent confusion and inefficient use.*	Role of the professional, item 10: The healthcare professional as the starting point	​
**Question**: Are the responsibilities and roles of healthcare professionals clearly defined in an implementation plan for the DHT? Explain how this has been shaped and how this is communicated to the healthcare professional.		
*Clear and timely communication about the DHT between the different layers in the hospital (for example, between the board of directors and the staff) contributes to the satisfaction of healthcare professionals and prevents resistance. It is important that healthcare professionals are kept informed about developments around DHT, so that they are involved and remain motivated. The head nurse or another department head can play a key role by filtering relevant information and sharing it with the rest of the department.*	Communication, item 11: Communication between hospital layers	​
**Question**: Are healthcare professionals informed in a timely and appropriate manner about developments and the further process surrounding the DHT? Explain how communication is structured and how it is ensured that the right information reaches the healthcare professional.		
*Training is essential to reduce resistance to DHT. Healthcare professionals, especially those with less experience, benefit from targeted training that facilitates the use of the technology. In-person training, manuals and videos contribute to a smooth learning process and ensure that healthcare professionals can use the technology effectively. Training should also be included in the onboarding process for new employees and be offered during working hours.*	Training, item 12: Providing training	​
**Question**: Is sufficient training available for healthcare professionals to learn to use DHT effectively? Explain how the training is offered, which resources are used, at what times the training takes place, and how such training is also included in the onboarding process.		
*Regularly evaluating the training methods to assess whether they still align well with practice can help to test whether healthcare professionals feel sufficiently competent in using the DHT. This evaluation can provide timely adjustments and additional support where needed.*	Training, item 13: Evaluating training	​
**Question**: Are periodic evaluations carried out to assess whether healthcare professionals feel competent in using DHT? Explain how these evaluations are conducted, what feedback is collected, and how this input is used to refine further training.		
*It is important that care providers develop specific skills, both technical (such as operating the technology) and medical (such as recognizing problems in remote monitoring). Distinguishing between these different types of skills, and steering training accordingly, ensures that healthcare professionals can use the technology effectively and respond appropriately in clinical situations.*	Training, item 14: Acquiring skills	​
**Question***:* Is the education/training for DHT well aligned to both technical and medical skills? Explain how the education is structured and how the different types of skills (technical and medical) are addressed.		

*DHT: digital health technology.* *Note.* Items are intended as prompts to document how the implementation addresses each aspect. The right-handed column can be used to document how each item is addressed in the implementation process. **Bold** indicates the actionable prompt (Question) for each checklist item. *Italics *indicate the explanatory rationale accompanying each checklist item.

## Discussion

In this study we developed a reflective tool to support systematic consideration of implementation conditions of DHTs on HCPs. Building on a re-analysis of existing literature, priority-setting among stakeholders, and qualitative exploration, six themes were identified: acceptance, champions, process and development, role of the professional, communication, and training. These themes were integrated into a practical tool designed to address a key gap in the field. While existing frameworks describe determinants of DHT implementation, they provide limited guidance on how to structurally incorporate HCPs’ perspectives into decision-making processes. Although our tool focuses on implementation-related factors, it is important to distinguish these from intrinsic technology characteristics, such as usability, which require separate, complementary evaluation approaches.

Our results align with and extend implementation science. Acceptance was described not as a static attitude but as a trajectory: professionals weigh early time costs (e.g., training) against credible long-term gains. This is consistent with literature that adoption depends on perceived usefulness and workflow fit, beyond usability alone.^15,35,36^ A related mechanism is the workload paradox: a DHT designed to reduce workload may shift tasks and intensify monitoring or clerical burden, particularly early in implementation, ultimately creating a higher workload.^15^ Such shifts are well-documented around electronic health records and are associated with burnout and lower professional satisfaction.^37^ This mechanism is consistent with the Job Demands–Resources model, which posits that elevated workload constitutes a job demand associated with negative outcomes for professional well-being.^38^ These insights highlight the need to address shifting workload demands early, to support both effective adoption and professional well-being.

Participants described marked variation in implementation policies and processes across hospitals. Some hospitals centralised innovation within specialist teams, whereas others devolved responsibility to individuals within departments, yielding variability and duplicated effort. Participants expressed a preference for a coordinated ‘single-front-door’ model, in analogy to central ethics committees approving research protocols across hospitals. Participants proposed standardised, transferable criteria for core domains (e.g., legal compliance, safety, technical requirements) to accelerate upscaling. Embedding the tool within coordinated governance could help ensure that workforce considerations are routinely considered alongside patient outcomes. This is important, because patient outcomes and cost-effectiveness dominate considerations in the implementation process, and workforce outcomes remain implicit or absent.^39^

Our findings highlight a conspicuous blind spot for the impact of DHTs on HCP well-being. During our focus-groups, HCPs found it difficult to reflect on what impact DHTs had on them, as they tend to prioritise patient satisfaction over their own well-being. A striking finding was the difficulty participants had in articulating impacts on their own well-being, even when explicitly asked. This could suggest that workforce outcomes remain largely implicit in digital transformation, reflecting cultural and organisational barriers to discussing well-being at all. It is also therefore, that we were unable to incorporate well-being outcomes into the checklist, and remain with implementation conditions that can be of influence on the well-being of the HCP. International guidance has begun to stress routine monitoring and evaluation of DHTs, including the impact of DHT on work and workforce.^40^ However, evaluations of HCP well-being rarely use standardised measures, as a standardised outcome set that together represents a construct of HCP well-being, is lacking, which leaves evaluations to ad-hoc measures and limits comparability across projects. Without an agreed framework for what to measure, targeted policy responses remain challenging. Developing a concise set of well-being indicators, could provide the foundation for cumulative learning and earlier detection of unintended harms or benefits. Work towards such a set is underway and may enable a more consistent assessment in the future.^41^

### Strengths and limitations

A key strength of this study is the integration of multiple data sources into a coherent development process. The collaborative, multi-institutional setting also enhanced the relevance of the findings for our institutions. Limitations include the small survey sample size and limited participation of actively practicing HCPs in our qualitative steps, potentially restricting the bandwidth of perspectives captured. Our study must therefore be seen as exploratory, and as a first step towards structural inclusion of the impact of DHT on the HCP, and furthermore of HCPs’ perspectives in DHT-implementation. The limited sample may as well reflect the current status of DHT-implementation, where the HCP is often overlooked. Finally, the tool has not yet been validated, and its feasibility, reliability, and impact remain to be evaluated. It should therefore be viewed as a starting point, rather than a final product.

### Practical implications and future research

In practical terms, the tool prompts teams to assess workload implications before implementation, to identify credible peer champions early, to embed training into onboarding structures, to ensure role clarity within implementation plans, and to establish feedback loops during deployment. These steps can be integrated into existing governance structures such as DHT committees or innovation desks. The checklist can be used at multiple decision points in DHT-implementation as a process tool for responsible implementation. Funders could request its inclusion with business cases, covering a brief note to better understand possible implications of the DHT-implementation on the HCP. Hospital boards or programme leads can use the checklist at key stages to consider communication routes, and onboarding needs of involved HCPs. Implementation teams and department leads could co-design with the HCP, confirm appropriate champions across disciplines, and adjust workflows where technology risks adding burden. Training should be embedded into onboarding pathways to ensure consistency and role clarity. Next steps would include piloting the checklist within ongoing implementation programmes to assess feasibility, followed by refinement through structured feedback. Furthermore, the reliability of interpretation across users and whether checklist-informed implementation planning is associated with improved implementation processes (e.g., better training uptake) and with routine monitoring of workforce outcomes using complementary measures (e.g., job satisfaction) should be evaluated. Multi-site and international testing will be important to assess transferability across contexts.

## Conclusion

This mixed-methods study developed a 14-item reflective checklist that identifies where implementation conditions could impact HCPs. By synthesising literature, integrating stakeholder priorities, and exploring qualitative insights, we derived six themes that translate professional considerations into practical prompts for use during implementation, and post-deployment monitoring. The checklist offers a pragmatic approach to incorporate implementation conditions that influence HCP well-being into DHT-implementation. Future work should test the tool in real-world settings, align it with standardised workforce outcome measures, and evaluate whether its use improves implementation processes and mitigates unintended workload and engagement harms.

## Supplemental material

Supplemental material - Towards responsible digital health implementation: A mixed-methods exploratory study developing a tool to assess workforce experience Supplemental material for Towards responsible digital health implementation: A mixed-methods exploratory study developing a tool to assess workforce experience by Bazuin Tom, Michiel Sebastiaan Oerbekke, Pauline Heus, Mike Petrus Theodora Kusters, Dave Anton Dongelmans, Annelies Visser, Arie Franx, Johann Klaus Götz Wietasch, Lotty Hooft and van der Laan Maarten Jurriaan in Digital Health.

Supplemental material - Towards responsible digital health implementation: A mixed-methods exploratory study developing a tool to assess workforce experience Supplemental material for Towards responsible digital health implementation: A mixed-methods exploratory study developing a tool to assess workforce experience by Bazuin Tom, Michiel Sebastiaan Oerbekke, Pauline Heus, Mike Petrus Theodora Kusters, Dave Anton Dongelmans, Annelies Visser, Arie Franx, Johann Klaus Götz Wietasch, Lotty Hooft and van der Laan Maarten Jurriaan in Digital Health.

## Data Availability

The data that support the findings of this study are available from the corresponding author, upon reasonable request.*

## ORCID iDs

Bazuin Tom https://orcid.org/0009-0001-9134-9704

Michiel Sebastiaan Oerbekke https://orcid.org/0000-0002-1861-3046

Pauline Heus https://orcid.org/0000-0002-6886-4652

Mike Petrus Theodora Kusters https://orcid.org/0009-0009-6178-1465

Dave Anton Dongelmans https://orcid.org/0000-0001-8477-6671

Annelies Visser https://orcid.org/0000-0001-7102-2627

Arie Franx https://orcid.org/0000-0001-8801-5546

Johann Klaus Götz Wietasch https://orcid.org/0000-0001-8001-5648

Lotty Hooft https://orcid.org/0000-0002-7950-2980

Maarten Jurriaan van der Laan https://orcid.org/0000-0002-3125-1342
