# Supplementary material for: Towards responsible digital health implementation: A mixed-methods exploratory study developing a tool to assess workforce experience
Source: Digit Health. 2026 Jul 6;12:20552076261450419. doi: 10.1177/20552076261450419 (PMC13338532; doi:10.1177/20552076261450419)
Supplement: Supplemental material - Towards responsible digital health implementation: A mixed-methods exploratory study developing a tool to assess workforce experience [file sj-pdf-1-dhj-10.1177_20552076261450419.pdf]

Towards Responsible Digital Health Implementation: A Mixed-Methods Exploratory Study Developing a Tool to Assess Workforce Experience

Supplementary material

Search strategy scoping review – Text S1 2

Analytic process of re-analysis of scoping review – Text S2 4

Full survey (Dutch and English) – Text S3 6

Topic list interviews and focus groups – Text S4 19

Good Reporting of A Mixed Methods Study (GRAMMS) checklist – Text S5 23

Preferred Reporting Items for Systematic reviews and Meta-Analyses extension for Scoping Reviews (PRISMA-ScR) Checklist – Text S6 24

Consolidated criteria for reporting qualitative studies (COREQ): 32-item checklist – Text S7 26

Included studies of original scoping review -Table S1 28

Reference List Original Scoping Review -Text S8 36

Study selection flow diagram – Figure S1 47

Reasons for exclusion – Table S2 48

Themes found in re-analysis of scoping review – Tables S3a and S3b, Figure S2 51

Ranking of themes according to the Reweighted Priority-Setting tool – Table S4 55

Ranking of themes, unweighted sum score, sensitivity check– Table S5 56

SEARCH STRATEGY SCOPING REVIEW – TEXT S1

Search strategy Embase

No.	Query	Results
#1	wearable computer'/exp OR 'medical informatics'/exp OR 'electronic health record'/exp OR 'artificial intelligence'/exp OR 'mobile application'/exp OR 'digital technology'/exp OR 'telehealth'/exp	283811
#2	((digital NEXT/1 health):ti) OR mhealth:ti OR ehealth:ti OR telehealth:ti OR telemedicine:ti OR ((wearable NEXT/1 device*):ti) OR ((mobile NEXT/1 health):ti) OR ((health NEXT/1 apps):ti) OR ((artificial NEXT/1 intelligence):ti) OR 'electronic health record*':ti OR 'electronic medical record*':ti OR 'health data analytics':ti OR 'health information technology':ti OR (((virtual OR augmented) NEAR/2 reality):ti) OR blockchain:ti OR 'decision support system*':ti OR ((remote NEAR/2 (monitoring OR sensing)):ti) OR chatbot*:ti OR ((digital NEAR/2 therapeutic*):ti) OR gamification:ti OR 'consumer health informatics':ti	99275
#3	#1 OR #2	319886
#4	hospital'/exp	1511753
#5	tertiary care center'/exp	118216
#6	secondary care center'/exp	2517
#7	hospital department'/exp	28458
#8	hospital:ti,ab,kw OR hospitals:ti,ab,kw OR icu:ti,ab,kw OR 'in hospital':ti,ab,kw OR inpatient*:ti,ab,kw OR 'emergency department':ti,ab,kw OR 'emergency departments':ti,ab,kw OR 'in patient':ti,ab,kw OR ward:ti,ab,kw OR wards:ti,ab,kw OR (((tertiary OR secondary) NEXT/1 (health OR instit* OR hospital* OR center* OR centre*)):ti,ab,kw)	2838989
#9	#4 OR #5 OR #6 OR #7 OR #8	3270339
#10	#3 AND #9	60908
#11	framework*:ti OR predictor*:ti OR indicator*:ti OR ((success NEAR/2 factor*):ti) OR barrier*:ti OR facilitator*:ti OR determinant*:ti OR characteristic*:ti OR model:ti OR models:ti OR protocol*:ti OR element:ti OR elements:ti OR concept:ti OR concepts:ti	1862719
#12	#10 AND #11	5335
#13	evaluat*:ti,ab,kw OR implement*:ti,ab,kw	7144513
#14	#12 AND #13	2787
#15	#14 NOT 'conference abstract'/it	2048
#16	#15 AND [embase]/lim	1556
#17	((review OR study) NEXT/1 protocol):ti	23488
#18	#16 NOT #17	1338

Search strategy MEDLINE

No.	Query	Results
1	exp *Telemedicine/ or exp *Digital Technology/ or *gamification/ or *mobile applications/ or exp *Artificial Intelligence/ or exp *Electronic Health Records/ or exp *Medical Informatics/ or exp *Wearable Electronic Devices/ or (digital adj health).ti. or mhealth.ti. or ehealth.ti. or telehealth.ti. or telemedicine.ti. or (wearable adj device*).ti. or (mobile adj health).ti. or (health adj apps).ti. or (artificial adj intelligence).ti. or "electronic health record*".ti. or "electronic medical record*".ti. or "health data analytics".ti. or "health information technology".ti. or ((virtual or augmented) adj2 reality).ti. or blockchain.ti. or "decision support system*".ti. or (Remote adj2 (Monitoring or sensing)).ti. or chatbot*.ti. or (Digital adj2 Therapeutic*).ti. or gamification.ti. or "Consumer Health Informatics".ti.	447996
2	exp "Hospitals"/ or exp "Home Care Services, Hospital-Based"/ or exp "Tertiary Care Centers"/ or exp "Secondary Care Centers"/ or exp "Hospital Departments"/ or (hospital or hospitals or ICU or in-hospital or inpatient* or emergency-department or emergency-departments or in-patient or ward or wards or ((tertiary or secondary) adj (health or instit* or hospital or hospitals or center or centers or centre or centres))).ti,ab,kf.	1977688
3	1 and 2	45957
4	(framework* or predictor* or indicator* or (success adj2 factor*) or barrier* or facilitator* or determinant* or characteristic* or (model or models) or protocol* or (element or elements) or (concept or concepts)).ti.	1512698
5	3 and 4	4097
6	(evaluat* or implement*).ti,ab,kf.	5157093
7	5 and 6	2049
8	((review or study) adj protocol).ti.	23145
9	7 not 8	1842

ANALYTIC PROCESS OF RE-ANALYSIS OF SCOPING REVIEW – TEXT S2

We documented our analytic process and modifications to the Work-System Model to transparently show how determinants relevant to healthcare professionals (HCPs) were structured. From the included studies, we extracted quotes describing barriers or facilitators for HCPs during digital health technology (DHT) implementation. Using inductive, semantic coding, these quotes were translated into descriptive labels; multiple labels could be assigned to a single quote when more than one determinant was present.

Next, labels were mapped onto a modified Work-System Model to capture determinants within a broader sociotechnical work system. Because many determinants explicitly related to patients (e.g., patient skills, engagement, or effects on HCP–patient communication), we added "Patient" as a separate domain to the original model. In addition, we operationalised interactions between domains as explicit mapping targets (e.g., DHT–Task, Organisation–People, HCP–Patient), because a substantial proportion of determinants described misfits or dependencies across domains rather than within one domain alone.

After mapping, we performed reflexive thematic analysis within each domain and within each interaction set, iteratively clustering labels into candidate themes and refining them through repeated comparison with the underlying quotes. This process resulted in themes and subthemes where appropriate, which are presented per domain.

FULL SURVEY (DUTCH AND ENGLISH) – TEXT S3

Survey 2 digitale zorgtechnologie

Record ID ______________________________

Geachte deelnemer,

Het Universitair Medisch Centrum Groningen (UMCG) onderzoekt samen met de Nederlandse Federatie van UMC's (NFU) en het Zorginstituut Nederland (ZIN) de impact van digitale zorgtechnologieën (afgekort tot DHT op zorgprofessionals.

In een eerdere, uitgebreide vragenlijst hebben we verschillende factoren gepresenteerd en gevraagd om aanvullingen. In deze nieuwe vragenlijst hebben we een aantal wijzigingen doorgevoerd. We zijn heden vooral benieuwd naar de factoren waarvan wij vinden dat ze impact hebben op (het welzijn van) de zorgprofessional en welke prioriteit deze verdienen bij de evaluatie van digitale zorgtechnologie (afgekort DHT).

De lijst bestaat uit 3 delen: een deel met algemene vragen, een deel met vragen over prioritering en een deel met enkele afsluitende vragen.

Het invullen van de vragenlijst duurt ongeveer 10 minuten. Deelname is vrijwillig. Ingevulde gegevens zullen vertrouwelijk worden behandeld.

Alvast bedankt voor uw deelname!

Vriendelijke groet,

Tom Bazuin, arts-onderzoeker UMCG

Door hieronder uw akkoord te geven, bevestigt u dat u de bovenstaande informatie heeft gelezen en begrepen, en dat u vrijwillig instemt om deel te nemen aan deze survey.<br><br>Ik geef mijn toestemming om deel te nemen aan deze survey.	◯ Yes<br>◯ No

Deel 1/3 Algemene gegevens

Wat is uw leeftijd?	◯ 18 jaar of jonger<br>◯ 18 - 24 jaar<br>◯ 25 - 34 jaar<br>◯ 35 - 44 jaar<br>◯ 45 - 54 jaar<br>◯ 55 - 64 jaar<br>◯ 65 jaar of ouder<br>◯ Wil ik niet zeggen
Wat is uw geslacht?	◯ Vrouw<br>◯ Man<br>◯ Anders<br>◯ Wil ik niet zeggen

Welke optie omschijft uw huidige functie het beste?	◯ Zorgprofessional<br>◯ Manager<br>◯ Beleidsmaker<br>◯ Anders
Indien u bij de vorige vraag 'Anders' hebt aangevinkt, kunt u dat hier toelichten.	________________________________
Indien u bij het vorige antwoord 'Zorgprofessional' hebt aangevinkt, kunt hier dit nog verder specificeren?	◯ Verpleegkundige<br>◯ Verpleegkundig specialist<br>◯ Medisch specialist<br>◯ Arts-assistent<br>◯ Paramedicus<br>◯ Onderzoeker<br>◯ Anders
Indien u bij de vorige vraag 'Anders' hebt aangevinkt, kunt u hier uw antwoord verder toelichten.	________________________________
Hoeveel jaren werkt u al in de zorg?	◯ Minder dan 1 jaar<br>◯ 1 - 5 jaren<br>◯ 6 - 10 jaren<br>◯ 11- 20 jaren<br>◯ 20 jaren of langer
In welk type zorginstelling werkt u voornamelijk?	◯ Universitair ziekenhuis<br>◯ Streekziekenhuis<br>◯ Verpleeghuis<br>◯ Huisartspraktijk<br>◯ Thuiszorg
Wat is uw ervaring met digitale zorgtechnologie? (meerdere antwoorden mogelijk)	☐ Ontwikkeling<br>☐ Implementatie<br>☐ Gebruik<br>☐ Ondersteuning<br>☐ Anders
U heeft aangegeven op een andere manier ervaring te hebben met digitale zorgtechnologie. Licht dit toe.	________________________________
Hoeveel jaar ervaring heeft u met het gebruik van digitale zorgtechnologieën?	◯ Minder dan 1 jaar<br>◯ 1 - 3 jaar<br>◯ 4 - 6 jaar<br>◯ 7 - 10 jaar<br>◯ Meer dan 10 jaar
Hoe vaak gebruikt u digitale zorgtechnologieën in uw werk?	◯ Dagelijks<br>◯ Wekelijks<br>◯ Maandelijks<br>◯ Jaarlijks of minder

Wat is uw niveau van vaardigheid in het gebruik van digitale zorgtechnologieën als zorgverlener?

- ◯ Basis (Ik kan digitale zorgtechnologieën gebruiken, zoals het invoeren van gegevens in een patiëntportaal, maar ik vraag snel om hulp bij problemen).
- ◯ Gemiddeld (Ik ben in staat om zelfstandig digitale zorgtechnologieën te gebruiken en kan vaak zelf eenvoudige problemen oplossen wanneer deze zich voordoen).
- ◯ Gevorderd (Ik heb uitgebreide kennis van digitale zorgtechnologieën, integreer ze regelmatig, en raak niet snel in paniek bij technische problemen).
- ◯ Specialist (Ik heb training of werkervaring op het gebied van digitale zorgtechnologie, en ik ben in staat om complexe problemen op te lossen of anderen te begeleiden in het gebruik ervan).

Wat is het voornaamste type digitale zorgtechnologie waar u mee werkt? (denk aan telemedicine, online patiëntportaal, apps voor patiënten etc) NB. Wij zijn vooral benieuwd naar patiëntgerichte digitale zorgtechnologie. EPD valt hier niet onder.

____

Deel 2/3 Prioriteren

We willen u vragen om hieronder in de matrix aan te geven, welke prioriteit de getoonde thema's hebben wij de evaluatie van digitale zorgtechnologieën. 0 betekent geen prioriteit, 1 betekent lage prioriteit en 5 betekent hoge prioriteit.

Prioritering

	0 (geen prioriteit)	1 (lage prioriteit)	2	3	4	5 (hoe prioriteit)
Organisatie-support: de aanwezigheid van leiders en 'champions'	◯	◯	◯	◯	◯	◯
Organisatie-support: het betrekken van de juiste stakeholders	◯	◯	◯	◯	◯	◯
Acceptatie en gebruik van de DHT	◯	◯	◯	◯	◯	◯
Voordelen van gebruik van DHT	◯	◯	◯	◯	◯	◯
Collega's en communicatie: het betrekken van zorgprofessionals bij de ontwikkeling	◯	◯	◯	◯	◯	◯
Collega's en communicatie: leiderschap onder zorgprofessionals	◯	◯	◯	◯	◯	◯
Collega's en communicatie: negatieve interacties tussen zorgverleners	◯	◯	◯	◯	◯	◯

Collega's en communicatie: positieve interacties tussen zorgverleners	○	○	○	○	○	○
Competentie	○	○	○	○	○	○
Ervaringen met DHT: positieve ervaringen	○	○	○	○	○	○
Ervaringen met DHT: negatieve ervaringen	○	○	○	○	○	○
Ervaringen met DHT: ambivalente ervaringen	○	○	○	○	○	○
Angsten, overtuigingen en zorgen: over werk en werkdruk	○	○	○	○	○	○
Angsten, overtuigingen en zorgen: over patiënten, in relatie tot gebruik van DHT	○	○	○	○	○	○
Angsten, overtuigingen en zorgen: positieve gedachten en overtuigingen over DHT	○	○	○	○	○	○
Angsten, overtuigingen en zorgen: angst en onzekerheid van de zorgprofessional over DHT	○	○	○	○	○	○
Angsten, overtuigingen en zorgen: privacy, beveiliging en veiligheid	○	○	○	○	○	○
Gezondheid van de zorgverlener	○	○	○	○	○	○
Perceptie van de zorgverlener: dat DHT een last is	○	○	○	○	○	○
Perceptie van de zorgverlener: dat DHT gemakkelijk te gebruiken is	○	○	○	○	○	○
Perceptie van de zorgverlener: dat DHT iets toevoegt	○	○	○	○	○	○
Perceptie van de zorgverlener: over voorkeuren van de zorgverlener	○	○	○	○	○	○
Perceptie van de zorgverlener: over besef van aanwezigheid van DHT	○	○	○	○	○	○
Perceptie van de zorgverlener: over het nut van DHT	○	○	○	○	○	○
Training, onderwijs en ondersteuning	○	○	○	○	○	○

Training, onderwijs en ondersteuning: vaardigheden	○	○	○	○	○	○
De persoonlijkheidskenmerken van de zorgprofessional: bevlogenheid	○	○	○	○	○	○
De persoonlijkheidskenmerken van de zorgprofessional: open staan voor verandering	○	○	○	○	○	○
De persoonlijkheidskenmerken van de zorgprofessional: de mening van de zorgprofessional	○	○	○	○	○	○
De persoonlijkheidskenmerken van de zorgprofessional: weerstand	○	○	○	○	○	○
De werkdruk: hogere werkdruk bij DHT	○	○	○	○	○	○
De werkdruk: lagere of normale werkdruk bij DHT	○	○	○	○	○	○
Houding: houding van professional richting technologie	○	○	○	○	○	○
Houding: gebruikersgereedheid	○	○	○	○	○	○
Interprofessioneel samenwerken: samenwerking en teamdynamiek	○	○	○	○	○	○
Interprofessioneel samenwerken: communicatie tussen organisatie en professional	○	○	○	○	○	○
Interprofessioneel samenwerken: faciliteren van interacties, door organisatie	○	○	○	○	○	○
Organisatie en proces: betrekken van professional	○	○	○	○	○	○
Organisatie en proces: duidelijkheid rondom rol van professional	○	○	○	○	○	○
Organisatie en proces: hulp van 'seniors'	○	○	○	○	○	○
Verloop van communicatie tussen patiënt en professional	○	○	○	○	○	○
De patiënt-professional relatie	○	○	○	○	○	○
Verandering in workflow door DHT	○	○	○	○	○	○
Omgaan met veranderde werkdruk	○	○	○	○	○	○

3/3 Afsluiting

U kunt hier opmerkingen toevoegen over de eerder ingevulde items.	________________________________
Ik geef toestemming om op een later moment benaderd te worden voor mogelijke deelname aan een focusgroep.	◯ Yes<br>◯ No
Vul hier uw e-mailadres in om op een later moment benaderd te worden.	__________________________

Survey 2 Digital health technology

Impact of digital health technologies on the healthcare professional

Dear participant,

The University Medical Center Groningen (UMCG), together with the Dutch Federation of University Medical Centers (NFU) and the Dutch National Health Care Institute (ZIN), is studying the impact of digital health technologies (abbreviated as **DHT**) on healthcare professionals.

In an earlier, extensive questionnaire we presented various factors and asked for additions. In this new questionnaire we have made a number of changes. Today we are particularly interested in the factors that you believe affect the impact on the healthcare professional and the priority these deserve in the evaluation of digital health technologies (abbreviated as DHT).

The questionnaire consists of three parts: a section with general questions, a section with questions on prioritisation, and a section with a few closing questions.

Completing the questionnaire takes approximately 10 minutes. Participation is voluntary. The data you provide will be treated confidentially.

Thank you in advance for your participation!

Kind regards,

Tom Bazuin, physician-researcher, UMCG

Consent

By giving your agreement below, you confirm that you have read and understood the information above and that you voluntarily agree to participate in this survey.

I give my consent to participate in this survey.

☐ Yes ☐ No

Part 1/3 General information

What is your age? (select one)

☐ 18 years or younger

☐ 18–24 years

☐ 25–34 years

☐ 35–44 years

☐ 45–54 years

☐ 55–64 years

☐ 65 years or older

☐ Prefer not to say

What is your gender? (select one)

☐ Woman

☐ Man

☐ Other

☐ Prefer not to say

Which option best describes your current role/position? (select one)

☐ Healthcare professional

☐ Manager

☐ Policy maker

☐ Other

If you selected “Other”, please specify: ______________________________

If you selected “Healthcare professional”, how would you further specify your role? (select one)

☐ Nurse

☐ Nurse specialist (advanced practice nurse)

☐ Medical specialist

☐ Resident physician (trainee)

☐ Paramedic / allied health professional

☐ Researcher

☐ Other

If you selected “Other”, please specify: ______________________________

How many years have you worked in healthcare? (select one)

☐ Less than 1 year

☐ 1–5 years

☐ 6–10 years

☐ 11–20 years

☐ 20 years or more

In what type of healthcare organisation do you mainly work? (select one)

☐ University hospital

☐ General/regional hospital

☐ Nursing home

☐ General practice

☐ Home care

What is your experience with digital health technology? (multiple answers possible)

☐ Development

☐ Implementation

☐ Use

☐ Support

☐ Other

If “Other”, please describe: ____________________________

How many years of experience do you have using digital health technologies? (select one)

☐ Less than 1 year

☐ 1–3 years

☐ 4–6 years

☐ 7–10 years

☐ More than 10 years

How often do you use digital health technologies in your work? (select one)

☐ Daily

☐ Weekly

☐ Monthly

☐ Yearly or less

What is your level of proficiency in using digital health technologies as a healthcare provider? (select one)

☐ Basic (I can use digital health technologies, such as entering data in a patient portal, but I quickly ask for help when problems arise.)

☐ Intermediate (I am able to use digital health technologies independently and can often solve simple problems myself when they occur.)

☐ Advanced (I have extensive knowledge of digital health technologies, can solve complex problems, integrate them regularly, and do not easily panic when technical problems occur.)

☐ Specialist (I have training or experience in the field of digital health technology, and I am able to solve complex problems or guide others in using them.)

What is the main type of digital health technology you work with?

(e.g., telemedicine, online patient portal, apps for patients, etc.)

Note: We are mainly interested in **patient-oriented** digital health technology. The electronic patient record/EHR is **not included** here.

Answer: ____________________________

Part 2/3 Prioritisation

We would like to ask you to indicate what priority the themes shown have in the evaluation of digital health technologies.

0 = no priority, 1 = low priority, 5 = high priority (select one per row).

Themes to rate (0–5):

- Organisational support: Presence of leaders and 'champions'
- Organisational support: Involvement of appropriate stakeholders
- Acceptance and use of DHTs by healthcare professionals
- Perceived benefits of DHT use by healthcare professionals
- Colleagues and communication: Involvement of healthcare professionals in the development process
- Colleagues and communication: Leadership among healthcare professionals
- Colleagues and communication: Negative interactions between healthcare professionals

- Colleagues and communication: Positive interactions between healthcare professionals
- Competence of the healthcare professional
- Experiences with DHT: Positive experiences of the healthcare professional with DHT
- Experiences with DHT: Negative experiences of the healthcare professional with DHT
- Experiences with DHT: Ambivalent experiences of the healthcare professional with DHT
- Fears, beliefs and concerns: about work and workload
- Fears, beliefs and concerns: about patients, in relation to DHT use
- Fears, beliefs and concerns: positive thoughts and beliefs about DHT
- Fears, beliefs and concerns: the healthcare professional’s fear and uncertainty about DHT
- Fears, beliefs and concerns: privacy, security and safety
- Health of the healthcare professional
- Provider perception: that DHT is a burden
- Provider perception: that DHT is easy to use
- Provider perception: that DHT adds value
- Provider perception: the provider’s preferences
- Provider perception: awareness of the presence of DHT
- Provider perception: the usefulness of DHT
- Training, education and support
- Training, education and support: skills
- Healthcare professional personality traits: work engagement
- Healthcare professional personality traits: openness to change
- Healthcare professional personality traits: the professional’s opinion
- Healthcare professional personality traits: resistance
- Workload: higher workload with DHT
- Workload: lower or normal workload with DHT
- Attitude: The healthcare professional’s attitude toward the technology
- Attitude: user readiness
- Interprofessional collaboration: Collaboration and team dynamics

- Interprofessional collaboration: Communication between organisation and healthcare professionals
- Interprofessional collaboration: The organization facilitating interactions
- Organisation and process: Involvement of health-care professionals in the implementation process
- Organisation and process: Clarity regarding the role of health-care professionals in the process
- Organisation and process: Support from ‘seniors’ in the process
- How communication between patient and professional unfolds
- The patient–professional relationship
- Changes in workflow due to DHT
- Coping with changed workload

Part 3/3 Closing

You can add comments here about the items you answered earlier:

Comments: ______________________________

I give permission to be contacted at a later time for possible participation in a focus group.

☐ Yes ☐ No

Please enter your email address to be contacted at a later time: ___________________

TOPIC LIST INTERVIEWS AND FOCUS GROUPS – TEXT S4

General questions for each item

- What, in your view, is the importance of item X during the implementation or evaluation of digital health technology?
- How do you demonstrate that item X is sufficiently safeguarded?
    - When is it sufficient?
    - And who is responsible?

Themes

Benefits of using DHT as perceived by the healthcare professional

"*DHT should be seen as beneficial for the professional*."

- How does recognising the benefits of the digital health technology contribute to its implementation? Or: In your view, what is the importance of recognising the benefits of the technology?
- How do you demonstrate that the healthcare professional sufficiently recognises the benefits of using the technology?
    - And who is responsible for this?
    - When is this sufficient?

Acceptance by the healthcare professional and use of the DHT

"*The professional must accept the technology and show the intention to use it when it is implemented*."

- In your view, what is the importance of the professional's acceptance during the implementation or evaluation of digital health technology?
- How can you demonstrate that the healthcare professional accepts the technology and intends to use it?
    - And who is responsible for this?

- When is this sufficient?

Involving the healthcare professional in the process (organisational level)

"*The healthcare professional should be involved by the organisation throughout the entire digital health technology process.*"

- In your view, what is the importance of involving healthcare professionals in the implementation process at the organisational level?
- How can you demonstrate that healthcare professionals are sufficiently involved?
  - And who is responsible for this?
  - When is this sufficient?

The presence of leaders and "champions"

"*Within an organisation, having early leaders/champions, people who are enthusiastic and can pull the team forward, is important*."

- In your view, what is the importance of having early leaders or champions when implementing technology?
- How do you demonstrate that the presence of these early leaders is ensured?
  - And who is responsible for this?
  - When is this sufficient?

Involving the healthcare professional in development (individual level)

"*The healthcare professional should also be included at the individual level in the development of technology.*"

- In your view, what is the importance of involving the healthcare professional in the development of digital health technology?
- How can you demonstrate that healthcare professionals are sufficiently involved at the individual level in technology development?

    - And who is responsible for this?
    - When is this sufficient?

Involving the right stakeholders

“*All stakeholders should be involved in the process*.”

- In your view, what is the importance of involving the right stakeholders during the process?
- How can you demonstrate that stakeholder involvement has been sufficient?
    - And who is responsible for this?
    - When is this sufficient?

Training, education, and support

“*Training or education is essential for the healthcare professional to know how to handle technology*.”

- In your view, what is the importance of training and education for the healthcare professional when using technology?
- How do you demonstrate that training and education are sufficiently ensured?
    - And who is responsible for this?
    - When is this sufficient?

Training, education, and support: skills

“*Sometimes specific skills are needed to use technology*.”

- What is the importance of developing specific skills for using technology?
- How can you demonstrate that healthcare professionals have sufficiently developed these skills? Or: How do you demonstrate that the development of these skills is sufficiently ensured?
    - And who is responsible for this?
    - When is this sufficient?

Communication between the organisation and the healthcare professional

"*For optimal use, good communication from the organisation about the technology is important*."

- What is the importance of good communication about the technology between the organisation and the healthcare professional?
- How can you demonstrate that this communication is sufficient or has been adequately considered?
    - And who is responsible for this?
    - When is this sufficient?

Clarity regarding the healthcare professional's role in the process

"*The professional's role throughout the process should be clear. Which tasks does the professional have, what is expected, etc.?*"

- In your view, what is the importance of clarity regarding the healthcare professional's role in the process?
- How can you demonstrate that there is sufficient clarity about the professional's role?
    - And who is responsible for this?
    - When is this sufficient?

GOOD REPORTING OF A MIXED METHODS STUDY (GRAMMS) CHECKLIST – TEXT S5

Guideline	Section: page
Describe the justification for using a mixed methods approach to the research question.	Introduction – Page 5<br>Methods – Page 6-7
Describe the design in terms of the purpose, priority and sequence of methods	Methods – Page 6-10
Describe each method in terms of sampling, data collection and analysis	Methods – Page 6-10
Describe where integration has occurred, how it has occurred and who has participated in it	Methods – Page 10<br>Results – Page 16
Describe any limitation of one method associated with the present of the other method	Discussion – Page 22-23
Describe any insights gained from mixing or integrating methods	Discussion – Page 22-24

O'Cathain A, Murphy E, Nicholl J. The quality of mixed methods studies in health services research. J Health Serv Res Policy. 2008;13(2):92-98.

PREFERRED REPORTING ITEMS FOR SYSTEMATIC REVIEWS AND META-ANALYSES EXTENSION FOR SCOPING REVIEWS (PRISMA-SCR) CHECKLIST – TEXT S6

SECTION	ITEM	PRISMA-ScR CHECKLIST ITEM	REPORTED ON PAGE #
**TITLE**			
**Title**	1	Identify the report as a scoping review.	NA; building on ScR
**ABSTRACT**			
**Structured summary**	2	Provide a structured summary that includes (as applicable): background, objectives, eligibility criteria, sources of evidence, charting methods, results, and conclusions that relate to the review questions and objectives.	NA; building on ScR
**INTRODUCTION**			
**Rationale**	3	Describe the rationale for the review in the context of what is already known. Explain why the review questions/objectives lend themselves to a scoping review approach.	Description of mixed methods approach; page 6-7
**Objectives**	4	Provide an explicit statement of the questions and objectives being addressed with reference to their key elements (e.g., population or participants, concepts, and context) or other relevant key elements used to conceptualize the review questions and/or objectives.	Page 5-6
**METHODS**			
**Protocol and registration**	5	Indicate whether a review protocol exists; state if and where it can be accessed (e.g., a Web address); and if available, provide registration information, including the registration number.	NA
**Eligibility criteria**	6	Specify characteristics of the sources of evidence used as eligibility criteria (e.g., years considered, language, and publication status), and provide a rationale.	Page 6-7
**Information sources***	7	Describe all information sources in the search (e.g., databases with dates of coverage and contact with authors to identify additional sources), as well as the date the most recent search was executed.	Supplementary material
**Search**	8	Present the full electronic search strategy for at least 1 database, including any limits used, such that it could be repeated.	Supplementary material
**Selection of sources of evidence†**	9	State the process for selecting sources of evidence (i.e., screening and eligibility) included in the scoping review.	Page 6-7
**Data charting process‡**	10	Describe the methods of charting data from the included sources of evidence (e.g., calibrated forms or forms that have been tested by the team before their use, and whether data charting was done independently or in duplicate) and any processes for obtaining and confirming data from investigators.	Page 6-7
**Data items**	11	List and define all variables for which data were sought and any assumptions and simplifications made.	Page 6-7
**Critical appraisal of individual sources**	12	If done, provide a rationale for conducting a critical appraisal of included sources of evidence; describe the	NA

**of evidence§**		methods used and how this information was used in any data synthesis (if appropriate).	
**Synthesis of results**	13	Describe the methods of handling and summarizing the data that were charted.	Page 6-7
**RESULTS**			
**Selection of sources of evidence**	14	Give numbers of sources of evidence screened, assessed for eligibility, and included in the review, with reasons for exclusions at each stage, ideally using a flow diagram.	Page 11, supplementary material
**Characteristics of sources of evidence**	15	For each source of evidence, present characteristics for which data were charted and provide the citations.	Supplementary material
**Critical appraisal within sources of evidence**	16	If done, present data on critical appraisal of included sources of evidence (see item 12).	NA
**Results of individual sources of evidence**	17	For each included source of evidence, present the relevant data that were charted that relate to the review questions and objectives.	Supplementary material
**Synthesis of results**	18	Summarize and/or present the charting results as they relate to the review questions and objectives.	Supplementary material
**DISCUSSION**			
**Summary of evidence**	19	Summarize the main results (including an overview of concepts, themes, and types of evidence available), link to the review questions and objectives, and consider the relevance to key groups.	NA; main findings of whole study discussed page 21
**Limitations**	20	Discuss the limitations of the scoping review process.	NA; main limitations page 22-23
**Conclusions**	21	Provide a general interpretation of the results with respect to the review questions and objectives, as well as potential implications and/or next steps.	NA; general conclusion page 23-24
**FUNDING**			
**Funding**	22	Describe sources of funding for the included sources of evidence, as well as sources of funding for the scoping review. Describe the role of the funders of the scoping review.	Page 25

JBI = Joanna Briggs Institute; PRISMA-ScR = Preferred Reporting Items for Systematic reviews and Meta-Analyses extension for Scoping Reviews.

* Where *sources of evidence* (see second footnote) are compiled from, such as bibliographic databases, social media platforms, and Web sites.

† A more inclusive/heterogeneous term used to account for the different types of evidence or data sources (e.g., quantitative and/or qualitative research, expert opinion, and policy documents) that may be eligible in a scoping review as opposed to only studies. This is not to be confused with *information sources* (see first footnote).

‡ The frameworks by Arksey and O'Malley (6) and Levac and colleagues (7) and the JBI guidance (4, 5) refer to the process of data extraction in a scoping review as data charting.

§ The process of systematically examining research evidence to assess its validity, results, and relevance before using it to inform a decision. This term is used for items 12 and 19 instead of "risk of bias" (which is more applicable to systematic reviews of interventions) to include and acknowledge the various sources of evidence that may be used in a scoping review (e.g., quantitative and/or qualitative research, expert opinion, and policy document).

From: Tricco AC, Lillie E, Zarin W, O'Brien KK, Colquhoun H, Levac D, et al. PRISMA Extension for Scoping Reviews (PRISMAScR): Checklist and Explanation. Ann Intern Med. 2018;169:467–473. doi: 10.7326/M18-0850.

CONSOLIDATED CRITERIA FOR REPORTING QUALITATIVE STUDIES (COREQ): 32-ITEM CHECKLIST – TEXT S7

Developed from:
Tong A, Sainsbury P, Craig J. Consolidated criteria for reporting qualitative research (COREQ): a 32-item checklist for interviews and focus groups. International Journal for Quality in Health Care. 2007. Volume 19, Number 6: pp. 349 – 357

Item No	Guide Questions/Description	Reported (on Page #)
Domain 1: Research team and reflexivity		
Personal Characteristics		
1. Interviewer/ facilitator	Which author/s conducted the interview or focus group?	TB and MO; page 9
2. Credentials	What were the researcher's credentials? E.g., PhD, MD	TB: MD; MO: PhD
3. Occupation	What was their occupation at the time of the study?	Both researcher; page 9
4. Gender	Was the researcher male or female?	Both male
5. Experience and training	What experience or training did the researcher have?	TB had little experience with conducting qualitative research himself, MO had done qualitative research previously
Relationship with participants		
6. Relationship established	Was a relationship established prior to study commencement?	Partly. Participants were approached by mail before having the interview or focus group.
7. Participant knowledge of the interviewer	What did the participants know about the researcher? e.g. personal goals, reasons for doing the research?	Goals of research.
8. Interviewer characteristics	What characteristics were reported about the interviewer/facilitator? e.g. Bias, assumptions, reasons and interests in the research topic	Background.
Domain 2: study design		
Theoretical framework		
9. Methodological orientation and Theory	What methodological orientation was stated to underpin the study? e.g. grounded theory, discourse analysis, ethnography, phenomenology, content analysis	Braun & Clarke's reflexive thematic analysis, conducted from a contextualist/interpretivist perspective; page 9
Participant selection		
10. Sampling	How were participants selected? e.g., purposive, convenience, consecutive, snowball	Purposive and snowball sampling; page 9
11. Method of approach	How were participants approached? e.g., face-to-face, telephone, mail, email	Combination. Face-to-face, mail.; page 9
12. Sample size	How many participants were in the study?	10; page 13
13. Non-participation Setting	How many people refused to participate or dropped	None.

Item No	Guide Questions/Description	Reported (on Page #)
	out? Reasons?	
14. Setting of data collection	Where was the data collected? e.g., home, clinic, workplace	Workplace, online.; page 9
15. Presence of nonparticipants	Was anyone else present besides the participants and researchers?	No.
16. Description of sample	What are the important characteristics of the sample? e.g. demographic data, date	Profession; page 13
Data collection		
17. Interview guide	Were questions, prompts, and guides provided by the authors? Was it pilot tested?	Yes, and yes; page 9
18. Repeat interviews	Were repeat interviews carried out? If yes, how many?	No
19. Audio/visual recording	Did the research use audio or visual recording to collect the data?	Yes, video.; page 9
20. Field notes	Were field notes made during and/or after the interview or focus group?	Yes, by both TB and MO
21. Duration	What was the duration of the interviews or focus group?	60-120 minutes.; page 9
22. Data saturation	Was data saturation discussed?	NA
23. Transcripts returned	Were transcripts returned to participants for comment and/or correction?	No
Domain 3: analysis and findings		
Data analysis		
24. Number of data coders	How many data coders coded the data?	One; page 9
25. Description of the coding tree	Did the authors provide a description of the coding tree?	Yes; page 9
26. Derivation of themes	Were themes identified in advance or derived from the data?	Derived from data; page 9
27. Software	What software, if applicable, was used to manage the data?	NA
28. Participant checking	Did participants provide feedback on the findings?	No
Reporting		
29. Quotations presented	Were participant quotations presented to illustrate the themes/findings? Was each quotation identified? e.g., participant number	Yes and yes. Page 13-15
30. Data and findings consistent	Was there consistency between the data presented and the findings?	Yes.
31. Clarity of major themes	Were major themes clearly presented in the findings?	Yes. Page 12-16
32. Clarity of minor themes	Is there a description of diverse cases or a discussion of minor themes?	Yes. Page 15-16

INCLUDED STUDIES OF ORIGINAL SCOPING REVIEW -TABLE S1

S5 Table. *Individual study baseline characteristics of the original scoping review.*

First author (year)	Study design	Sample size	Country	Setting	WHO classification of digital health interventions category	WHO classification of digital health interventions sub-category	DHT development phase
Alarabyat (2023) (32)	Qualitative, exploratory study	24	Jordan	Major hospitals (n = 2)	2. Digital health interventions for healthcare providers	12. Telemedicine	Implementation
Alboraire (2021) (33)	Cross-sectional quantitative survey (Likert-/VAS-scale questions) with a convenience sampling method	686	Egypt	University teaching hospitals (n=8)	1. Digital health interventions for persons	12. Telemedicine	Implementation
AlDossary (2017) (34)	Framework development	NA	NA	Community setting	1. Digital health interventions for persons	12. Telemedicine	Development
Almojaibel (2021) (35)	Cross-sectional quantitative survey (Likert-scale questions) with a convenience sampling method	134	USA, Indiana	Pulmonary rehabilitation centers in hospitals of the Indiana State University (n = 8)	1. Digital health interventions for persons	12. Telemedicine	Implementation
Auret (2022) (36)	Mixed-methods: Quantitative survey (Likert-scale questions) and qualitative interview	47 (n= 10 interviews)	Australia	Telehealth clinics within the Great Southern hematology clinics	1. Digital health interventions for persons	12. Telemedicine	Implementation
Avdagovska (2020) (37)	Scoping review	NA	NA	Unclear (patient portals mainly used in hospitals)	1. Digital health interventions for persons	4. Personal health tracking	Implementation
Ayanlade (2019) (38)	Mixed methods study	300	Nigeria	Tertiary hospitals (n = 6)	1. Digital health interventions for persons	1. Targeted communication to Persons	Implementation
Azam (2023) (39)	Quantitative study	214	Pakistan	Public and private hospitals (n = 6)	1. Digital health interventions for persons	2. Untargeted communication to Persons	Implementation
Bagot (2020) (40)	Mixed methods study	Survey: 290<br>Interview: 11	Australia	Regional hospitals (n = 16)	2. Digital health interventions for healthcare providers	12. Telemedicine	Implementation
Bazzano (2018) (41)	Interpretive study using qualitative data	20	USA, Louisiana	Major health care systems (n = 3) and Federally Qualified Health Centers (n = 4)	2. Digital health interventions for healthcare providers	12. Telemedicine	Implementation

Bele (2021) (42)	Qualitative study	42	Canada	Tertiary (n =1) and regional hospital (n = 1)	2. Digital health interventions for healthcare providers	12. Telemedicine	Implementation
Berry (2023) (43)	quantitative and qualitative data.	19	USA	Hospital/emergency department	2. Digital health interventions for healthcare providers	12. Telemedicine	Implementation
Bonet (2023) (44)	Mixed-methods qualitative study.	26	USA, Texas	Hospital	2. Digital health interventions for healthcare providers	12. Telemedicine	Implementation
Burgess (2017) (45)	Qualitative (semi structured interviews)	10	Canada	Tertiary care hospital	1. Digital health interventions for persons	12. Telemedicine	Implementation
Busetto (2022) (46)	qualitative multi-method study	26	Germany	Regional stroke network, hospitals.	2. Digital health interventions for healthcare providers	12. Telemedicine	Implementation
Castor (2023) (47)	Qualitative interview study	10	Sweden	University hospital	2. Digital health interventions for healthcare providers	12. Telemedicine	Implementation
Catapan (2022) (48)	Exploratory case study (qualitative data)	NR	Brazil	University hospital and specialized municipal center	2. Digital health interventions for healthcare providers	12. Telemedicine	Implementation
Chen (2021) (49)	Quantitative survey (closed-ended questions)	5814	USA	Rural and urban area, general medical and surgery hospital-level	1. Digital health interventions for persons	12. Telemedicine	Implementation
Chen (2022) (50)	Qualitative (semi structured interviews); focus groups	64	China	Chinese healthcare system	1. Digital health interventions for persons	12. Telemedicine	Implementation
Cherniwchan (2022) (51)	Literature review	NA	NA	Inpatient palliative care or hospice palliative care	2. Digital health interventions for healthcare providers	12. Telemedicine	Implementation
Chiang (2015) (52)	Qualitative study, interviews	31	Taiwan	Hospitals	2. Digital health interventions for healthcare providers	12. Telemedicine	Implementation
Clark (2023) (53)	Multiple methods program evaluation, among which interviews with healthcare providers	Survey: 85<br>Interview: 6	Canada	Pregnancy Clinic	2. Digital health interventions for healthcare providers	12. Telemedicine	Implementation
Cunha (2023) (54)	Systematic Review	NA	USA	In-hospital	2. Digital health interventions for healthcare providers	12. Telemedicine	Implementation
Cwintal (2023) (55)	Rapid review (interviews; surveys; focus groups; design sessions; prototype evaluation)	NA	NR	Pediatric surgery	1. Digital health interventions for persons	Other	Development
De Benedictis (2019) (56)	Qualitative (survey)	191	Italy	University hospital	2. Digital health interventions for healthcare providers	13. Healthcare provider communication	Implementation

De Guzman (2020) (57)	Systematic Review	NA	NA	NR	2. Digital health interventions for healthcare providers	12. Telemedicine	Implementation
de Jong (2018) (58)	Proof of concept	20	Netherlands	Internal Medicine and Dermatology departments of a university medical center	1. Digital health interventions for persons	12. Telemedicine	Implementation
de Souza (2017) (59)	Qualitative (semi structured interviews)	28	Brazil	High technology hospital and remote healthcare units	2. Digital health interventions for healthcare providers	12. Telemedicine	Implementation
Deighton (2021) (60)	Prospective non-randomized cluster trial	53	UK	Hospital (cardiology ward and the acute assessment unit)	1. Digital health interventions for persons	12. Telemedicine	Implementation
Fang (2018) (61)	Mixed methods study	Survey: 89<br>Focus group: 49	USA	Community hospital and regional hospital	2. Digital health interventions for healthcare providers	12. Telemedicine	Implementation
Faulds (2021) (62)	Focus group	9	USA	Medical intensive care unit	1. Digital health interventions for persons	4. Personal health tracking	Implementation
Fleet (2022) (63)	Description of a model to telehealth implementation	NA	USA	Children Hospital Boston	2. Digital health interventions for healthcare providers	12. Telemedicine	Implementation
Fulop (2023) (64)	Mixed methods study	292	England	Hospital, remote home monitoring	NA/Unclear	Other	Implementation
Fulton (2022) (65)	Mixed methods study	Pre-implementation survey: 19<br>Post-implementation survey: 16	Australia	Mixed medical ward in hospital	1. Digital health interventions for persons	4. Personal health tracking	Implementation
Garg (2021) (66)	Prospective cohort study	22	India	Outpatient Movement Disorders clinic of the Neurology department at a tertiary level referral center	1. Digital health interventions for persons	12. Telemedicine	Implementation
Gesell (2018) (67)	Qualitative study, interviews	30	USA	Academic medical centers (n = 4)	2. Digital health interventions for healthcare providers	9. Identification and registration of persons	Implementation
Gore (2024) (68)	Qualitative (semi structured interviews)	20	Australia	Hospital and/or GP care visits (shared care)	1. Digital health interventions for persons	12. Telemedicine	Implementation
Gutierrez (2021) (69)	Mixed methods study	NR	USA	Tertiary hospital	2. Digital health interventions for healthcare providers	12. Telemedicine	Implementation
Gvozdanovic (2022) (70)	Proof of concept	6	UK	National Hospital for Neurology and Neurosurgery	1. Digital health interventions for persons	12. Telemedicine	Implementation
Habib (2023) (71)	Quantitative survey (Likert-scale questions)	163	Saudi Arabia	University Hospital	2. Digital health interventions for healthcare providers	12. Telemedicine	Implementation
Haddad (2021) (72)	Describing of organizational	NA	USA	Hospital	2. Digital health interventions for	12. Telemedicine	Implementation

	framework				healthcare providers		
Hall (2022) (73)	Expert panel meeting	13	Canada	Emergency department	2. Digital health interventions for healthcare providers	12. Telemedicine	Implementation
Hansen (2022) (74)	Multidisciplinary stakeholder panel	24	USA	Rural surgery	1. Digital health interventions for persons	Other	Implementation
Hilker (2023) (75)	Narrative review	NA	NA	ICU	2. Digital health interventions for healthcare providers	12. Telemedicine	Implementation
Holden (2016) (76)	Testing of an expanded technology assessment model	167	USA	Pediatric ICU (PICU)	2. Digital health interventions for healthcare providers	10. Person-centered health records	Implementation
Houlding (2021) (77)	Rapid Review	NA	NA	Unclear	2. Digital health interventions for healthcare providers	12. Telemedicine	Implementation
Hsiao (2019) (78)	Quantitative survey study (Likert-scale questions)	201	Taiwan	Hospital	2. Digital health interventions for healthcare providers	12. Telemedicine	Implementation
Hübner (2015) (79)	Proof of concept	Unclear (114 forms; 6 nurses; 14 patients)	Germany	Hospitals and nursing homes and ambulant nursing services	2. Digital health interventions for healthcare providers	10. Person-centered health records	Multiple
Huilgol (2020) (80)	Database analysis (predictor evaluation)	870	USA	Hospitals	1. Digital health interventions for persons	12. Telemedicine	Implementation
Indraratna (2022) (81)	Mixed methods: qualitative, process evaluation	NA	Australia	Hospital and GP	2. Digital health interventions for healthcare providers	12. Telemedicine	Implementation
Jaeger (2024) (82)	Pilot study of TeleEMS program	NR	USA	Academic medical center	2. Digital health interventions for healthcare providers	12. Telemedicine	Clinical validation/pilot (with patients)
Kahn (2019) (83)	An Ethnographic Study (using both quantitative and qualitative data)	Interviews: 222 Number of focus groups: 18	USA	US hospitals	2. Digital health interventions for healthcare providers	12. Telemedicine	Implementation
Kelley (2020) (84)	Qualitative analysis of case studies	25	Canada	Hospitals, home care and primary care	2. Digital health interventions for healthcare providers	12. Telemedicine	Implementation
Kgasi (2023) (85)	Quantitative approach, survey with close-ended questions.	158	South Africa	District hospitals	2. Digital health interventions for healthcare providers	12. Telemedicine	Implementation
Koivunen (2024) (86)	Systematic Review	NA	Multiple	Hospital setting, home care and outpatient clinic.	2. Digital health interventions for healthcare providers	12. Telemedicine	Implementation
Kooij (2018) (87)	Qualitative interviews	21	Netherlands	Hospitals (2 UMC's, 3 teaching and 2 general)	1. Digital health interventions for persons	4. Personal health tracking	Implementation
Korot (2022) (88)	Mixed methods: cohort and (qualitative) survey	417	UK	Eye hospital	1. Digital health interventions for persons	12. Telemedicine	Implementation

	study						
Koshy (2019) (89)	Quantitative survey (Likert-scale questions)	363	Australia	Tertiary hospitals	1. Digital health interventions for persons	4. Personal health tracking	Implementation
Kujala (2020) (90)	Qualitative interview study	10	Finland	Hospitals (n = 5)	2. Digital health interventions for healthcare providers	12. Telemedicine	Implementation
Lamprinos (2016) (91)	Framework development	NA	Multiple (Germany, Turkey)	University hospital, and GP	2. Digital health interventions for healthcare providers	12. Telemedicine	Clinical validation/pilot (with patients)
Lawrence (2023) (92)	Retrospective feasibility, acceptability, safety and impact study	331	Australia	Hospital level care at home	1. Digital health interventions for persons	12. Telemedicine	Implementation
Lin (2022) (93)	Mixed-methods: prospective study of the implementation of a novel internet-based family-centered care, using focus groups, interviews and survey data.	Focus group: 5<br>Implementation trial: 46<br>Postimplementation: 28	USA	Hospital (pediatrics)	1. Digital health interventions for persons	4. Personal health tracking	Implementation
Makhlouf (2023) (94)	Cross-sectional study (structured phone interviews)	40	Tunisia	Hospital (pediatric rheumatology department)	1. Digital health interventions for persons	12. Telemedicine	Exploration
Mercuri (2021) (95)	Framework development	NR	Italy	Hospital (pediatric care)	1. Digital health interventions for persons	12. Telemedicine	Development
Mora (2022) (96)	Describing of a protocol design	NA	Spain	Tertiary care hospital	2. Digital health interventions for healthcare providers	12. Telemedicine	Implementation
Morgan (2022) (97)	Quantitative survey (closed-ended questions)	164	USA	Rural academic medical center	1. Digital health interventions for persons	12. Telemedicine	Implementation
Mosch (2022) (98)	Qualitative study with interviews	7	Germany	ICU	NA/Unclear	Other	Implementation
Nguyen (2019) (99)	Phenomenological qualitative study using an interpretive description methodology	10	Canada	Rehabilitation hospital	1. Digital health interventions for persons	4. Personal health tracking	Implementation
Nouri (2020) (100)	Literature review	29	USA	Academic/community medical centers	1. Digital health interventions for persons	12. Telemedicine	Implementation
Nyoni (2023) (101)	Qualitative study, interviews	15	USA, Missouri	Rural hospitals (n = 3)	2. Digital health interventions for healthcare providers	12. Telemedicine	Implementation
O'Connor (2020) (102)	Observational quasi-experimental one	102	Canada	Hospital	2. Digital health interventions for	12. Telemedicine	Implementation

	group posttest-only design, quantitative survey data (Likert-scale questions)				healthcare providers		
Ofoma (2021) (103)	Cross-sectional database analysis	4396 hospitals (788 telemedicine critical care)	USA	Hospitals	1. Digital health interventions for persons	12. Telemedicine	Implementation
Ostervang (2019) (104)	Qualitative study	9	Denmark	Hospital (cancer department)	2. Digital health interventions for healthcare providers	12. Telemedicine	Implementation
Palacholla (2019) (105)	Scoping review	NA	Multiple	Primary care setting (n = 30/36)	2. Digital health interventions for healthcare providers	12. Telemedicine	Implementation
Paulsen (2019) (106)	Qualitative study with interviews and focus groups	27	Norway	University hospital	1. Digital health interventions for persons	4. Personal health tracking	Implementation
Pillay (2021) (107)	Quantitative, survey	147	South Africa	Hospitals, both urban and rural. different levels, among which tertiary.	2. Digital health interventions for healthcare providers	12. Telemedicine	Implementation
Pilosof (2021) (108)	Case study	40	Israel	Tertiary hospital	2. Digital health interventions for healthcare providers	12. Telemedicine	Implementation
Pineda (2023) (109)	Interview study	12	USA	Neonatal intensive care unit (NICU)	2. Digital health interventions for healthcare providers	12. Telemedicine	Implementation
Rakers (2023) (110)	Exploratory qualitative study (semi-structured interviews)	10	Netherlands	Hospitals	1. Digital health interventions for persons	12. Telemedicine	Implementation
Remmits (2024) (111)	Mixed methods study	102	Netherlands	Academic hospital (pediatric department)	2. Digital health interventions for healthcare providers	12. Telemedicine	Implementation
Rodrigues (2024) (112)	Systematic Review	NA	NA	Hospital and primary care	2. Digital health interventions for healthcare providers	17. Prescription and medication management	Implementation
Rosenthal (2022) (113)	Mixed methods study	246	USA	Level 1 pediatric trauma center	2. Digital health interventions for healthcare providers	12. Telemedicine	Implementation
Ruxwana (2014) (114)	Multiple qualitative case study	35	South Africa	Rural hospitals	1. Digital health interventions for persons	12. Telemedicine	Implementation
Sabesan (2018) (115)	description of implementation process and descriptive analysis.	62	Australia	Rural hospitals	2. Digital health interventions for healthcare providers	12. Telemedicine	Implementation
Sagaro (2020) (116)	Systematic Review	NA	Ethiopia	Hospital	2. Digital health interventions for healthcare providers	12. Telemedicine	Implementation

Schoville (2015) (117)	Theoretical review / creating implementation model.	NA	NA	Unspecified	NA/Unclear	Other	Implementation
Shaarani (2023) (118)	Cross sectional study with quantitative questionnaire	390	Lebanon	Hospitals and primary care	2. Digital health interventions for healthcare providers	12. Telemedicine	Implementation
Slevin (2019) (119)	Qualitative (semi structured interviews)	30	Ireland	University hospitals (n = 2)	1. Digital health interventions for persons	4. Personal health tracking	Exploration
Slevin (2020) (120)	Qualitative study, one-to-one semi structured interviews	32	Ireland	University hospitals (n = 2) and general practitioner clinics	NA/Unclear	Other	Implementation
Song (2021) (121)	Qualitative (semi structured interviews)	22	China	University hospital (Cardiology department)	1. Digital health interventions for persons	12. Telemedicine	Implementation
Steinberg (2022) (122)	Pilot prospective cohort study	24	USA	Tertiary care center (academic obstetrics and gynecology practices)	1. Digital health interventions for persons	12. Telemedicine	Implementation
Terry (2021) (123)	Quantitative, survey	136	USA	Rural Pennsylvania teaching hospital and associated satellite clinics	2. Digital health interventions for healthcare providers	12. Telemedicine	Implementation
Thomas (2022) (124)	Mixed method observational study design	80	Australia	Health departments in hospitals	2. Digital health interventions for healthcare providers	12. Telemedicine	Implementation
Tieu (2015) (125)	Qualitative (semi structured interviews)	16	USA	Safety net hospital	1. Digital health interventions for persons	12. Telemedicine	Exploration
Treskes (2019) (126)	Quantitative (survey study)	255	Netherlands	Hospitals	NA/Unclear	Other	Implementation
Tseng (2018) (127)	Case report	NA	NR	Academic medical centers	NA/Unclear	Other	Development
Turan (2021) (128)	Pilot study	468	Turkey	Hospital dermatology department	2. Digital health interventions for healthcare providers	12. Telemedicine	Implementation
Twamley (2024) (129)	Qualitative (semi structured focus groups)	13	UK	ICU	1. Digital health interventions for persons	6. On demand communication	Exploration
Valenta (2022) (131)	Mixed methods study	88	Multiple (Switzerland/Germany)	Hospital	2. Digital health interventions for healthcare providers	12. Telemedicine	Implementation
Valenta (2021) (130)	Description of framework development and testing	NA	USA	Academic hospital	2. Digital health interventions for healthcare providers	12. Telemedicine	Implementation
van den Wijngaart (2018) (132)	Qualitative survey study	51	Netherlands	Hospital	1. Digital health interventions for persons	4. Personal health tracking	Implementation
Verweij (2022) (133)	Qualitative (focus groups and interviews)	61	Netherlands	Academic and peripheral hospitals	1. Digital health interventions for persons	1. Targeted communication to Persons	Implementation

Wang (2023) (134)	Case study and quantitative survey (Likert-scale questions)	263	USA	Children’s hospital	1. Digital health interventions for persons	4. Personal health tracking	Implementation
Watt (2022) (135)	Interview study	20	Canada	Geriatric medicine clinic	2. Digital health interventions for healthcare providers	12. Telemedicine	Implementation
Weigel (2021) (136)	Case study of two models	NA	USA	Children’s hospital, critical care and Emergency department	2. Digital health interventions for healthcare providers	12. Telemedicine	Implementation
Weinstein (2014) (137)	Review	NA	USA	Unclear (includes hospitals among others)	NA/Unclear	12. Telemedicine	Implementation
Whitehead (2024) (138)	Qualitative (semi-structured interviews)	8	USA	Academic medical systems	2. Digital health interventions for healthcare providers	12. Telemedicine	Implementation
Whitelaw (2021) (139)	Systematic scoping review	NA	NA	Clinical practice	NA/Unclear	Other	Implementation
Yang (2022) (140)	Framework/model development	10	China	Secondary general and tertiary hospital	NA/Unclear	Other	Implementation
Yu (2022) (141)	Systematic Review and framework development	NA	Multiple	Healthcare system including hospitals	1. Digital health interventions for persons	12. Telemedicine	Implementation
Zachrison (2020) (142)	Quantitative survey (closed-ended questions)	527	USA	Rural emergency departments	1. Digital health interventions for persons	12. Telemedicine	Implementation
Zailani (2014) (143)	Literature review, model proposal and quantitative structured survey	117	Malaysia	Public hospitals	1. Digital health interventions for persons	12. Telemedicine	Implementation

Abbreviations: GP, general practice; ICU, intensive care unit; NA, not applicable; NR, not reported; UK, United Kingdom; UMC, university medical center; US(A), United States (of America)

Reference list.

REFERENCE LIST ORIGINAL SCOPING REVIEW -TEXT S8

32. Alarabyat IA, Al-Nsair N, Alrimawi I, Al-Yateem N, Shudifat RM, Saifan AR. Perceived barriers to effective use of telehealth in managing the care of patients with cardiovascular diseases: a qualitative study exploring healthcare professionals' views in Jordan. BMC Health Services Research. 2023;23(1):452.

33. Alboraie M, Allam MA, Youssef N, Abdalgaber M, El-Raey F, Abdeen N, et al. Knowledge, Applicability, and Barriers of Telemedicine in Egypt: A National Survey. Int J Telemed Appl. 2021;2021:5565652.

34. AlDossary S, Martin-Khan MG, Bradford NK, Armfield NR, Smith AC. The Development of a Telemedicine Planning Framework Based on Needs Assessment. J Med Syst. 2017;41(5):74.

35. Almojaibel AA, Munk N, Goodfellow LT, Fisher TF, Miller KK, Comer AR, et al. Determinants of Telerehabilitation Acceptance among Patients Attending Pulmonary Rehabilitation Programs in the United States. Saudi J Med Med Sci. 2021;9(3):230-4.

36. Auret K, Pikora T, Pola M. Specialist haematology consultation services in regional Western Australia: evaluating a model combining telehealth and onsite clinics. Intern Med J. 2022;52(3):451-7.

37. Avdagovska M, Menon D, Stafinski T. Capturing the impact of patient portals based on the quadruple aim and benefits evaluation frameworks: Scoping review. Journal of Medical Internet Research. 2020;22(12).

38. Ayanlade OS, Oyebisi TO, Kolawole BA. Health Information Technology Acceptance Framework for diabetes management. Heliyon. 2019;5(5):e01735.

39. Azam M, Bin Naeem S, Kamel Boulos MN, Faiola A. Modelling the Predictors of Mobile Health (mHealth) Adoption among Healthcare Professionals in Low-Resource Environments. Int J Environ Res Public Health. 2023;20(23).

40. Bagot K, Moloczij N, Arthurson L, Hair C, Hancock S, Bladin CF, et al. Nurses' Role in Implementing and Sustaining Acute Telemedicine: A Mixed-Methods, Pre-Post Design Using an Extended Technology Acceptance Model. Journal of Nursing Scholarship. 2020;52(1):34-46.

41. Bazzano AN, Wharton MK, Monnette A, Nauman E, Price-Haywood E, Glover C, et al. Barriers and facilitators in implementing non-face-to-face chronic care management in an elderly population with diabetes: A qualitative study of physician and health system perspectives. Journal of Clinical Medicine. 2018;7(11).

42. Bele S, Cassidy C, Curran J, Johnson DW, Bailey JAM. Using the Theoretical Domains Framework to Identify Barriers and Enablers to Implementing a Virtual Tertiary-Regional Telemedicine Rounding and

Consultation for Kids (TRaC-K) Model: Qualitative Study. Journal of Medical Internet Research. 2021;23(12):e28610.

43. Berry CA, Kwok L, Gofine M, Kaufman M, Williams DA, Terlizzi K, et al. Utilization and Staff Perspectives on an On-Demand Telemedicine Model for People with Intellectual and Developmental Disabilities Who Reside in Certified Group Residences. Telemedicine Reports. 2023;4(1):204-14.

44. Bonet O, Sasangohar F. A sociotechnical framework for integration of telehealth into clinical workflow. IISE Transactions on Healthcare Systems Engineering. 2023;13(3):248-59.

45. Burgess K, Atkinson KM, Westeinde J, Crowcroft N, Deeks SL, Wilson K. Barriers and facilitators to the use of an immunization application: a qualitative study supplemented with Google Analytics data. J Public Health (Oxf). 2017;39(3):e118-e26.

46. Busetto L, Sert M, Herzog F, Hoffmann J, Stang C, Amiri H, et al. "But it's a nice compromise" - Qualitative multi-centre study of barriers and facilitators to acute telestroke cooperation in a regional stroke network. European Journal of Neurology. 2022;29(1):208-16.

47. Castor C, Lindkvist RM, Hallstrom IK, Holmberg R. Health Care Professionals' Experiences and Views of eHealth in Pediatric Care: Qualitative Interview Study Applying a Theoretical Framework for Implementation. JMIR Pediatrics and Parenting. 2023;6:e47663.

48. Catapan SC, Taylor A, Calvo MCM. Health professionals' views of medical teleconsultation uptake in the Brazilian Unified Health System: A description using the NASSS framework. International Journal of Medical Informatics. 2022;168:104867.

49. Chen J, Amaize A, Barath D. Evaluating Telehealth Adoption and Related Barriers Among Hospitals Located in Rural and Urban Areas. J Rural Health. 2021;37(4):801-11.

50. Chen N. Stakeholder Power Analysis of the Facilitators and Barriers for Telehealth Solution Implementation in China: A Qualitative Study of Individual Users in Beijing and Interviews With Institutional Stakeholders. JMIR Form Res. 2022;6(1):e19448.

51. Cherniwchan HR. Harnessing New and Existing Virtual Platforms to Meet the Demand for Increased Inpatient Palliative Care Services During the COVID-19 Pandemic: A 5 Key Themes Literature Review of the Characteristics and Barriers of These Evolving Technologies. American Journal of Hospice & Palliative Medicine. 2022;39(5):591-7.

52. Chiang KF, Wang HH, Chien IK, Liou JK, Hung CL, Huang CM, et al. Healthcare providers' perceptions of barriers in implementing of home telecare in Taiwan: a qualitative study. International Journal of Medical Informatics. 2015;84(4):277-87.

53. Clark A, Jung E, Prusky C, Shah BR, Halperin IJ. An Evaluation of Virtual Care for Gestational Diabetes Using the Quadruple Aim Framework: Assessment of Patient and Provider Experience, Cost, and Clinical Outcomes. Canadian Journal of Diabetes. 2023;47(3):236-42.e3.

54. Cunha AS, Pedro AR, Cordeiro JV. Facilitators of and Barriers to Accessing Hospital Medical Specialty Telemedicine Consultations During the COVID-19 Pandemic: Systematic Review. J Med Internet Res. 2023;25:e44188.

55. Cwintal M, Ranjbar H, Bandamiri P, Guadagno E, Osmanlliu E, Poenaru D. A rapid review for developing a co-design framework for a pediatric surgical communication application. J Pediatr Surg. 2023;58(5):879-90.

56. De Benedictis A, Lettieri E, Masella C, Gastaldi L, Macchini G, Santu C, et al. WhatsApp in hospital? An empirical investigation of individual and organizational determinants to use. PLoS ONE [Electronic Resource]. 2019;14(1):e0209873.

57. De Guzman KR, Snoswell CL, Taylor ML, Senanayake B, Haydon HM, Batch JA, et al. A Systematic Review of Pediatric Telediabetes Service Models. Diabetes Technology & Therapeutics. 2020;22(8):623-38.

58. de Jong JM, Ogink PA, van Bunningen CG, Driessen RJ, Engelen LJ, Heeren B, et al. A Cloud-Based Virtual Outpatient Clinic for Patient-Centered Care: Proof-of-Concept Study. J Med Internet Res. 2018;20(9):e10135.

59. de Souza CHA, Morbeck RA, Steinman M, Hors CP, Bracco MM, Kozasa EH, et al. Barriers and Benefits in Telemedicine Arising Between a High-Technology Hospital Service Provider and Remote Public Healthcare Units: A Qualitative Study in Brazil. Telemedicine Journal & E-Health. 2017;23(6):527-32.

60. Deighton AJ, Davies C, Bourantas C, Knight C, Woldman S, Deighton J, et al. Investigating consultant-led virtual review as a model for implementing 7-day cardiology services in UK clinical practice. Future Healthc J. 2021;8(3):e666-e70.

61. Fang JL, Asiedu GB, Harris AM, Carroll K, Colby CE. A Mixed-Methods Study on the Barriers and Facilitators of Telemedicine for Newborn Resuscitation. Telemedicine Journal & E-Health. 2018;24(10):811-7.

62. Faulds ER, Jones L, McNett M, Smetana KS, May CC, Sumner L, et al. Facilitators and Barriers to Nursing Implementation of Continuous Glucose Monitoring (CGM) in Critically Ill Patients With COVID-19. Endocrine Practice. 2021;27(4):354-61.

63. Fleet SE, Davidson RD, Carr K, Lubenow C, Rouse AS, Truscott KE. Conversion of a Traditional In-Person Feeding Clinic to a Telehealth-Only Model of Care. Maternal & Child Health Journal. 2022;26(1):58-64.

64. Fulop NJ, Walton H, Crellin N, Georghiou T, Herlitz L, Litchfield I, et al. A rapid mixed-methods evaluation of remote home monitoring models during the COVID-19 pandemic in England. Health and Social Care Delivery Research. 2023;11(13):1-151.

65. Fulton S, Janssen H, Salih S, James A, Elphinston RA. Feasibility and acceptability of a mobile model of environmental enrichment for patients with mixed medical conditions receiving inpatient rehabilitation: A mixed methods study. BMJ Open. 2022;12(9).

66. Garg D, Majumdar R, Chauhan S, Preenja R, Parihar J, Saluja A, et al. Teleneurorehabilitation Among Person with Parkinson's Disease in India: The Initial Experience and Barriers to Implementation. Ann Indian Acad Neurol. 2021;24(4):536-41.

67. Gesell SB, Golden SL, Limkakeng AT, Carr CM, Matuskowitz A, Smith LM, et al. Implementation of the HEART Pathway: Using the consolidated framework for implementation research. Critical Pathways in Cardiology. 2018;17(4):191-200.

68. Gore C, Lisy K, O'Callaghan C, Wood C, Emery J, Martin A, et al. Colorectal cancer survivors' experiences and views of shared and telehealth models of survivorship care: A qualitative study. Psychooncology. 2024;33(1):e6265.

69. Gutierrez J, Moeckli J, Holcombe A, O'Shea AMJ, Bailey G, Rewerts K, et al. Implementing a Telehospitalist Program Between Veterans Health Administration Hospitals: Outcomes, Acceptance, and Barriers to Implementation. Journal of Hospital Medicine. 2021;16(3):156-63.

70. Gvozdanovic A, Jozsa F, Fersht N, Grover PJ, Kirby G, Kitchen N, et al. Integration of a personalised mobile health (mHealth) application into the care of patients with brain tumours: proof-of-concept study (IDEAL stage 1). BMJ Surg Interv Health Technol. 2022;4(1):e000130.

71. Habib S, Alsulaim KB, Mobeirek OA, Alsaeed AM, Albawardi FA, Alqahtani YK, et al. Barriers and Facilitators of Telemedicine Among Physicians at a University Hospital. Cureus. 2023;15(9):e45078.

72. Haddad TC, Blegen RN, Prigge JE, Cox DL, Anthony GS, Leak MA, et al. A Scalable Framework for Telehealth: The Mayo Clinic Center for Connected Care Response to the COVID-19 Pandemic. Telemedicine Reports. 2021;2(1):78-87.

73. Hall JN, Ackery AD, Dainty KN, Gill PS, Lim R, Masood S, et al. Designs, facilitators, barriers, and lessons learned during the implementation of emergency department led virtual urgent care programs in Ontario, Canada. Frontiers in Digital Health. 2022;4.

74. Hansen RN, Saour BM, Serafini B, Hannaford B, Kim L, Kohno T, et al. Opportunities and Barriers to Rural Telerobotic Surgical Health Care in 2021: Report and Research Agenda from a Stakeholder Workshop. Telemed J E Health. 2022;28(7):1050-7.

75. Hilker S, Mathias S, Raman D, Anand S, Brewster R, Britto C. Shared features of successful tele-ICU models–A narrative review of successful implementation with a focus on LMIC models. Health Policy and Technology. 2023;12(4).

76. Holden RJ, Asan O, Wozniak EM, Flynn KE, Scanlon MC. Nurses' perceptions, acceptance, and use of a novel in-room pediatric ICU technology: testing an expanded technology acceptance model. BMC Medical Informatics & Decision Making. 2016;16(1):145.

77. Houlding E, Mate KKV, Engler K, Ortiz-Paredes D, Pomey MP, Cox J, et al. Barriers to Use of Remote Monitoring Technologies Used to Support Patients With COVID-19: Rapid Review. JMIR MHealth and UHealth. 2021;9(4):e24743.

78. Hsiao JL, Chen RF. Understanding Determinants of Health Care Professionals' Perspectives on Mobile Health Continuance and Performance. JMIR Medical Informatics. 2019;7(1):e12350.

79. Hübner U, Schulte G, Sellemann B, Quade M, Rottmann T, Fenske M, et al. Evaluating a Proof-of-Concept Approach of the German Health Telematics Infrastructure in the Context of Discharge Management. Stud Health Technol Inform. 2015;216:492-6.

80. Huilgol YS, Miron-Shatz T, Joshi AU, Hollander JE. Hospital Telehealth Adoption Increased in 2014 and 2015 and Was Influenced by Population, Hospital, and Policy Characteristics. Telemed J E Health. 2020;26(4):455-61.

81. Indraratna P, Biswas U, Liu H, Redmond SJ, Yu J, Lovell NH, et al. Process Evaluation of a Randomised Controlled Trial for TeleClinical Care, a Smartphone-App Based Model of Care. Frontiers in Medicine. 2022;8.

82. Jaeger LR, McCartin MP, Haamid A, Weber JM, Tataris KL. TeleEMS: An EMS Telemedicine Pilot Program Barriers to Implementation. Prehospital Emergency Care. 2024;28(2):363-8.

83. Kahn JM, Rak KJ, Kuza CC, Ashcraft LE, Barnato AE, Fleck JC, et al. Determinants of Intensive Care Unit Telemedicine Effectiveness. An Ethnographic Study. American Journal of Respiratory & Critical Care Medicine. 2019;199(8):970-9.

84. Kelley LT, Fujioka J, Liang K, Cooper M, Jamieson T, Desveaux L. Barriers to Creating Scalable Business Models for Digital Health Innovation in Public Systems: Qualitative Case Study. JMIR Public Health and Surveillance. 2020;6(4):e20579.

85. Kgasi M, Chimbo B, Motsi L. mHealth Self-Monitoring Model for Medicine Adherence of Patients With Diabetes in Resource-Limited Countries: Structural Equation Modeling Approach. JMIR Formative Research. 2023;7:e49407.

86. Koivunen M, Saranto K. Nursing professionals' experiences of the facilitators and barriers to the use of telehealth applications: - A systematic review of qualitative evidence. JBI Database of Systematic Reviews and Implementation Reports. 2014;10(57):3894-906.

87. Kooij L, Groen WG, van Harten WH. Barriers and Facilitators Affecting Patient Portal Implementation from an Organizational Perspective: Qualitative Study. Journal of Medical Internet Research. 2018;20(5):e183.

88. Korot E, Pontikos N, Drawnel FM, Jaber A, Fu DJ, Zhang G, et al. Enablers and Barriers to Deployment of Smartphone-Based Home Vision Monitoring in Clinical Practice Settings. JAMA Ophthalmol. 2022;140(2):153-60.

89. Koshy AN, Ko J, Sajeev JK, Rajakariar K, Roberts L, Cooke J, et al. Evaluating patient attitudes and barriers towards smart technology for cardiac monitoring: results from a prospective multicentre study. BMJ Innovations. 2019;5(2-3):101-7.

90. Kujala S, Ammenwerth E, Kolanen H, Ervast M. Applying and Extending the FITT Framework to Identify the Challenges and Opportunities of Successful eHealth Services for Patient Self-Management: Qualitative Interview Study. Journal of Medical Internet Research. 2020;22(8):e17696.

91. Lamprinos I, Demski H, Mantwill S, Kabak Y, Hildebrand C, Ploessnig M. Modular ICT-based patient empowerment framework for self-management of diabetes: Design perspectives and validation results. International Journal of Medical Informatics. 2016;91:31-43.

92. Lawrence J, Truong D, Dao A, Bryant PA. Virtual hospital-level care-feasibility, acceptability, safety and impact of a pilot Hospital-In-The-Home model for COVID-19 infection. Front Digit Health. 2023;5:1068444.

93. Lin JL, Huber B, Amir O, Gehrmann S, Ramirez KS, Ochoa KM, et al. Barriers and Facilitators to the Implementation of Family-Centered Technology in Complex Care: Feasibility Study. Journal of Medical Internet Research. 2022;24(8).

94. Makhlouf Y, Nessib DB, Ferjani H, Triki W, Maatallah K, Dhia K, et al. The concept of telemedicine in pediatric rheumatology in Tunisia: Parents' perceptions. J Pediatr Nurs. 2023;69:6-9.

95. Mercuri E, Zampino G, Morsella A, Pane M, Onesimo R, Angioletti C, et al. Contactless: a new personalised telehealth model in chronic pediatric diseases and disability during the COVID-19 era. Ital J Pediatr. 2021;47(1):29.

96. Mora C, Sampedro I, Rodríguez-Caballero A, Martín-Láez R, Ortega-Roldán M, Venkatraghavan L, et al. Barriers and facilitators in the implementation of a telemedicine-based outpatient brain tumor surgery program. Neurosurg Focus. 2022;52(6):E8.

97. Morgan A, Goodman D, Vinagolu-Baur J, Cass I. Prenatal telemedicine during COVID-19: patterns of use and barriers to access. JAMIA Open. 2022;5(1):ooab116.

98. Mosch LK, Poncette A-S, Spies C, Weber-Carstens S, Schieler M, Krampe H, et al. Creation of an Evidence-Based Implementation Framework for Digital Health Technology in the Intensive Care Unit: Qualitative Study. JMIR Formative Research. 2022;6(4):e22866.

99. Nguyen AV, Ong YA, Luo CX, Thuraisingam T, Rubino M, Levin MF, et al. Virtual reality exergaming as adjunctive therapy in a sub-acute stroke rehabilitation setting: facilitators and barriers. Disability & Rehabilitation Assistive Technology. 2019;14(4):317-24.

100. Nouri SS, Adler-Milstein J, Thao C, Acharya P, Barr-Walker J, Sarkar U, et al. Patient characteristics associated with objective measures of digital health tool use in the United States: A literature review. J Am Med Inform Assoc. 2020;27(5):834-41.

101. Nyoni T, Evers EC, Perez M, Jeffe DB, Fritz SA, Colditz GA, et al. Perceived barriers and facilitators to the adoption of telemedicine infectious diseases consultations in southeastern Missouri hospitals. Journal of Telemedicine & Telecare. 2023:1357633X221149461.

102. O'Connor Y, Andreev P, O'Reilly P. MHealth and perceived quality of care delivery: a conceptual model and validation. BMC Medical Informatics & Decision Making. 2020;20(1):41.

103. Ofoma UR, Maddox TM, Perera C, Waken RJ, Drewry AM, Liu L, et al. Characteristics of U.S. Acute Care Hospitals That Have Implemented Telemedicine Critical Care. Crit Care Explor. 2021;3(7):e0468.

104. Ostervang C, Vestergaard LV, Dieperink KB, Danbjorg DB. Patient Rounds With Video-Consulted Relatives: Qualitative Study on Possibilities and Barriers From the Perspective of Healthcare Providers. Journal of Medical Internet Research. 2019;21(3):e12584.

105. Palacholla RS, Fischer N, Coleman A, Agboola S, Kirley K, Felsted J, et al. Provider- and Patient-Related Barriers to and Facilitators of Digital Health Technology Adoption for Hypertension Management: Scoping Review. JMIR Cardio. 2019;3(1):e11951.

106. Paulsen MM, Varsi C, Paur I, Tangvik RJ, Andersen LF. Barriers and Facilitators for Implementing a Decision Support System to Prevent and Treat Disease-Related Malnutrition in a Hospital Setting: Qualitative Study. JMIR Formative Research. 2019;3(2):e11890.

107. Pillay L, Govender R, Pillay S. Doctor-perceived-barriers to telephone clinics at KwaZulu-Natal hospitals during the COVID-19 pandemic. South African Family Practice. 2021;63(1):e1-e6.

108. Pilosof NP, Barrett M, Oborn E, Barkai G, Pessach IM, Zimlichman E. Inpatient Telemedicine and New Models of Care during COVID-19: Hospital Design Strategies to Enhance Patient and Staff Safety. International Journal of Environmental Research & Public Health [Electronic Resource]. 2021;18(16):08.

109. Pineda R, Smith D, Richter M, Gruskin BA, Dusing S, Peden CJ. Health Care Professionals' Perceptions About a Telehealth Model of Therapy After NICU Discharge. OTJR: Occupational Therapy Journal of Research. 2023;43(3):495-504.

110. Rakers MM, van Os HJA, Recourt K, Mosis G, Chavannes NH, Struijs JN. Perceived barriers and facilitators of structural reimbursement for remote patient monitoring, an exploratory qualitative study. Health Policy and Technology. 2023;12(1):100718.

111. Remmits AJW, van Mastrigt GAPG, Evers SMAA, van Setten PA. Facilitators and barriers to the transition from outpatient clinic visits to home-based check-ups for children being treated with growth hormone: a mixed-methods study. European Journal of Pediatrics. 2024.

112. Rodrigues DA, Roque M, Mateos-Campos R, Figueiras A, Herdeiro MT, Roque F. Barriers and facilitators of health professionals in adopting digital health-related tools for medication appropriateness: A systematic review. Digital Health. 2024;10:20552076231225133.

113. Rosenthal JL, Haynes SC, Bonilla B, Rominger K, Williams J, Sanders A, et al. Enhancing the Implementation of the Virtual Pediatric Trauma Center Using Practical, Robust, Implementation and Sustainability Model: A Mixed-Methods Study. Telemedicine Reports. 2022;3(1):137-48.

114. Ruxwana N, Herselman M, Pottas D. A Generic Quality Assurance Model (GQAM) for successful e-health implementation in rural hospitals in South Africa. Health Inf Manag. 2014;43(1):26-36.

115. Sabesan S, Senko C, Schmidt A, Joshi A, Pandey R, Ryan CA, et al. Enhancing Chemotherapy Capabilities in Rural Hospitals: Implementation of a Telechemotherapy Model (QReCS) in North Queensland, Australia. Journal of oncology practice/American Society of Clinical Oncology. 2018;14(7):e429-e37.

116. Sagaro GG, Battineni G, Amenta F. Barriers to Sustainable Telemedicine Implementation in Ethiopia: A Systematic Review. Telemedicine Reports. 2020;1(1):8-15.

117. Schoville RR, Titler MG. Guiding healthcare technology implementation: a new integrated technology implementation model. CIN: Computers, Informatics, Nursing. 2015;33(3):99-107; quiz E1.

118. Shaarani I, Jounblat M, Jounblat H, Ghanem A, Mansour R, Taleb R. Developing and Validating a Tool to Assess Telemedicine Acceptance Among Physicians During Pandemic Using a Technology Acceptance Model. Telemedicine Journal & E-Health. 2023;29(6):903-11.

119. Slevin P, Kessie T, Cullen J, Butler MW, Donnelly SC, Caulfield B. A qualitative study of chronic obstructive pulmonary disease patient perceptions of the barriers and facilitators to adopting digital health technology. Digit Health. 2019;5:2055207619871729.

120. Slevin P, Kessie T, Cullen J, Butler MW, Donnelly SC, Caulfield B. Exploring the barriers and facilitators for the use of digital health technologies for the management of COPD: a qualitative study of clinician perceptions. Qjm. 2020;113(3):163-72.

121. Song T, Liu F, Deng N, Qian S, Cui T, Guan Y, et al. A Comprehensive 6A Framework for Improving Patient Self-Management of Hypertension Using mHealth Services: Qualitative Thematic Analysis. J Med Internet Res. 2021;23(6):e25522.

122. Steinberg JR, Yeh C, Jackson J, Saber R, Niznik CM, Leziak K, et al. Optimizing Engagement in an mHealth Intervention for Diabetes Support During Pregnancy: the Role of Baseline Patient Health and Behavioral Characteristics. J Diabetes Sci Technol. 2022;16(6):1466-72.

123. Terry DL, Buntoro SP. Perceived Usefulness of Telehealth Among Rural Medical Providers: Barriers to Use and Associations with Provider Confidence. Journal of Technology in Behavioral Science. 2021;6(4):567-71.

124. Thomas EE, Taylor ML, Ward EC, Hwang R, Cook R, Ross JA, et al. Beyond forced telehealth adoption: A framework to sustain telehealth among allied health services. Journal of Telemedicine & Telecare. 2022:1357633X221074499.

125. Tieu L, Sarkar U, Schillinger D, Ralston JD, Ratanawongsa N, Pasick R, et al. Barriers and Facilitators to Online Portal Use Among Patients and Caregivers in a Safety Net Health Care System: A Qualitative Study. J Med Internet Res. 2015;17(12):e275.

126. Treskes RW, Wildbergh TX, Schalij MJ, Scherptong RWC. Expectations and perceived barriers to widespread implementation of e- Health in cardiology practice: Results from a national survey in the Netherlands. Netherlands Heart Journal. 2019;27(1):18-23.

127. Tseng J, Samagh S, Fraser D, Landman AB. Catalyzing healthcare transformation with digital health: Performance indicators and lessons learned from a Digital Health Innovation Group. Healthc (Amst). 2018;6(2):150-5.

128. Turan Ç, Utlu Z. Investigating the factors enabling the accurate implementation of the patient-assisted teledermatology model during the pandemic in Turkey: A pilot study. Dermatologic Therapy. 2021;34(2).

129. Twamley J, Hamer O, Hill J, Kenyon R, Twamley H, Casey R, et al. Exploring the perceptions of former ICU patients and clinical staff on barriers and facilitators to the implementation of virtual reality exposure therapy: A qualitative study. Nursing in Critical Care. 2024;29(2):313-24.

130. Valenta S, Harvey J, Sederstrom E, Glanville M, Walsh T, Ford D. Enterprise Adoption of Telehealth: An Academic Medical Center's Experience Utilizing the Telehealth Service Implementation Model. Telemedicine Reports. 2021;2(1):163-70.

131. Valenta S, Ribaut J, Leppla L, Mielke J, Teynor A, Koehly K, et al. Context-specific adaptation of an eHealth-facilitated, integrated care model and tailoring its implementation strategies-A mixed-methods study as a part of the SMILe implementation science project. Frontiers in Health Services. 2022;2:977564.

132. van den Wijngaart LS, Geense WW, Boehmer AL, Brouwer ML, Hugen CA, van Ewijk BE, et al. Barriers and Facilitators When Implementing Web-Based Disease Monitoring and Management as a Substitution for Regular Outpatient Care in Pediatric Asthma: Qualitative Survey Study. Journal of Medical Internet Research. 2018;20(10):e284.

133. Verweij L, Smit Y, Blijlevens NM, Hermens RP. A comprehensive eHealth implementation guide constructed on a qualitative case study on barriers and facilitators of the digital care platform CMyLife. BMC Health Services Research. 2022;22(1):751.

134. Wang EY, Kennedy KM, Zhang L, Qian D, Forbes T, Zuniga-Hernandez M, et al. Predicting pediatric healthcare provider use of virtual reality using a technology acceptance model. JAMIA Open. 2023;6(3):ooad076.

135. Watt JA, Fahim C, Straus SE, Goodarzi Z. Barriers and facilitators to virtual care in a geriatric medicine clinic: A semi-structured interview study of patient, caregiver and healthcare provider perspectives. Age and Ageing. 2022;51(1).

136. Weigel PA, Merchant KA, Wittrock A, Kissee J, Ullrich F, Bell AL, et al. Paediatric tele-emergency care: A study of two delivery models. Journal of Telemedicine & Telecare. 2021;27(1):23-31.

137. Weinstein RS, Lopez AM, Joseph BA, Erps KA, Holcomb M, Barker GP, et al. Telemedicine, telehealth, and mobile health applications that work: opportunities and barriers. Am J Med. 2014;127(3):183-7.

138. Whitehead DC, Li KY, Hayden E, Jaffe T, Karam A, Zachrison KS. Evaluating the Quality of Virtual Urgent Care: Barriers, Motivations, and Implementation of Quality Measures. Journal of General Internal Medicine. 2024.

139. Whitelaw S, Pellegrini DM, Mamas MA, Cowie M, Van Spall HGC. Barriers and facilitators of the uptake of digital health technology in cardiovascular care: a systematic scoping review. European Heart Journal Digital Health. 2021;2(1):62-74.

140. Yang J, Luo B, Zhao C, Zhang H. Artificial intelligence healthcare service resources adoption by medical institutions based on TOE framework. Digit Health. 2022;8:20552076221126034.

141. Yu J, de Antonio A, Villalba-Mora E. Design of an Integrated Acceptance Framework for Older Users and eHealth: Influential Factor Analysis. J Med Internet Res. 2022;24(1):e31920.

142. Zachrison KS, Boggs KM, Hayden EM, Espinola JA, Camargo CA, Jr. Understanding Barriers to Telemedicine Implementation in Rural Emergency Departments. Ann Emerg Med. 2020;75(3):392-9.

143. Zailani S, Gilani MS, Nikbin D, Iranmanesh M. Determinants of telemedicine acceptance in selected public hospitals in Malaysia: clinical perspective. J Med Syst. 2014;38(9):111.

STUDY SELECTION FLOW DIAGRAM – FIGURE S1

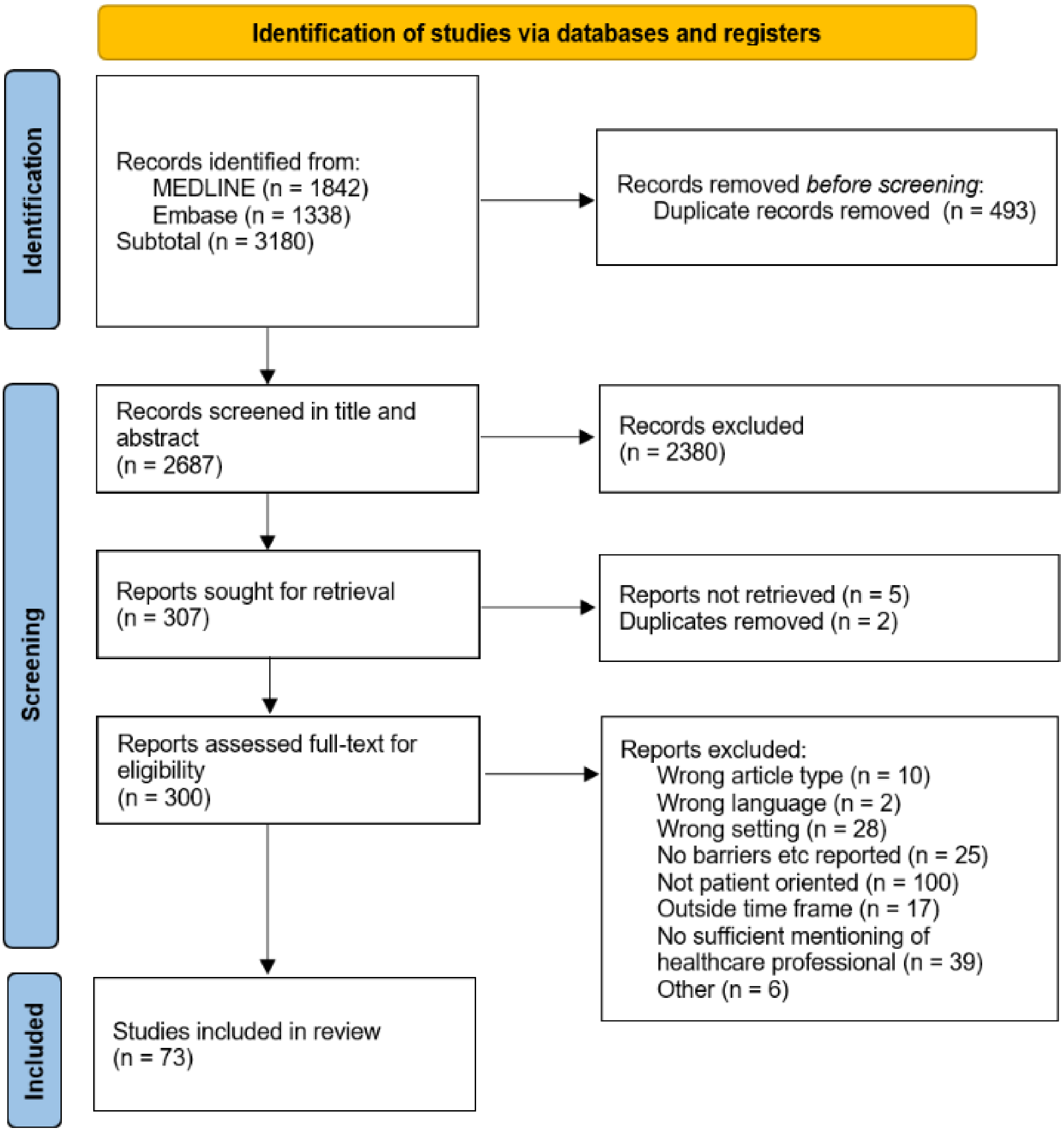

***Figure S1**: PRISMA flowchart*

REASONS FOR EXCLUSION – TABLE S2

Table S2. *Excluded studies, including reasons for exclusion, in the re-analyses of the scoping review by Kusters et al (submitted)*

Author	Year	Title	Reason for exclusion
Alboraire	2021	Knowledge, Applicability, and Barriers of Telemedicine in Egypt: A National Survey	no information on healthcare professionals
AlDossary	2017	The Development of a Telemedicine Planning Framework Based on Needs Assessment	no information on healthcare professionals
Almojaibel	2021	Determinants of Telerehabilitation Acceptance among Patients Attending Pulmonary Rehabilitation Programs in the United States	no information on healthcare professionals, aimed at patients
Auret	2022	Specialist haematology consultation services in regional Western Australia: evaluating a model combining telehealth and onsite clinics	no information on healthcare professionals, aimed at patients
Azam	2023	Modelling the Predictors of Mobile Health (mHealth) Adoption among Healthcare Professionals in Low-Resource Environments	no explicit barriers or facilitators reported, only determinants of adoption intention
Burgess	2017	Barriers and facilitators to the use of an immunization application: a qualitative study supplemented with Google Analytics data	no information on healthcare professionals, aimed at patients
Chen	2022	Stakeholder Power Analysis of the Facilitators and barriers for Telehealth Solution implementation in China: A Qualitative Study of Individual Users in Beijing and Interviews With Institutional Stakeholders	no reporting on effects/factors regarding healthcare professionals
Chen	2021	Evaluating Telehealth Adoption and Related barriers Among hospitals Located in Rural and Urban Areas	no reporting on effects/factors regarding healthcare professionals
Cunha	2023	Facilitators of and Barriers to Accessing Hospital Medical Specialty Telemedicine Consultations During the COVID-19 Pandemic: Systematic Review	no reporting on factors regarding healtchare professionals (For instance, 'better work-life balance' refers to patients with CF.
Cwintal	2023	A rapid review for developing a co-design framework for a pediatric surgical communication application	no information on healthcare professionals
de Jong	2018	A Cloud-Based Virtual Outpatient Clinic for Patient-Centered Care: Proof-of-concept Study	no information on healthcare professionals
Deighton	2021	Investigating consultant-led virtual review as a model for implementing 7-day cardiology services in UK clinical practice	no information on healthcare professionals
Garg	2021	Teleneurorehabilitation among person with parkinson's disease in India: The initial experience and barriers to implementation	no information on healthcare professionals
Gore	2024	Colorectal cancer survivors' experiences and views of shared and telehealth models of survivorship care: A qualitative study	no information on healthcare professionals, aimed at patient experiences
Gvozdanovic	2022	Integration of a personalised mobile health (mHealth) application into the care of patients with brain tumours: Proof-of-concept study (IDEAL stage 1)	no information on healthcare professionals, aimed at patient experiences
Hansen	2022	Opportunities and Barriers to Rural Telerobotic Surgical Health Care in 2021: Report and Research Agenda from a Stakeholder Workshop	no information on healthcare professional factors, rather general factors
Hubner	2015	Evaluating a Proof-of-concept Approach of the German Health Telematics Infrastructure in the Context of Discharge Management	no information on healthcare professionals

Huilgol	2020	Hospital Telehealth Adoption Increased in 2014 and 2015 and Was Influenced by Population, hospital, and Policy characteristics	no reporting on effects/factors regarding healthcare professionals
Korot	2022	Enablers and barriers to Deployment of Smartphone-Based Home Vision Monitoring in Clinical Practice Settings	no information on healthcare professionals
Koshy	2019	Evaluating patient attitudes and barriers towards smart technology for cardiac monitoring: Results from a prospective multicentre study	no information on healthcare professionals
Lawrence	2023	Virtual hospital-level care—feasibility, acceptability, safety and impact of a pilot hospital-In-The-Home model for COVID-19 infection	no information on healthcare professionals
Makhlouf	2023	The concept of telemedicine in pediatric rheumatology in Tunisia: Parents' perceptions	no information on healthcare professionals
Mercuri	2021	Contactless: a new personalised telehealth model in chronic pediatric diseases and disability during the COVID-19 era	no reporting on effects/factors regarding healthcare professionals
Mora	2022	Barriers and facilitators in the implementation of a telemedicine-based outpatient brain tumor surgery program	barriers and facilitators reported on process, not specifically related to healthcare professional
Morgan	2022	Prenatal telemedicine during COVID-19: Patterns of use and barriers to access	no information on healthcare professionals, aimed at patient experiences
Nouri	2020	Patient characteristics associated with objective measures of digital health tool use in the United States: A literature review	no information on healthcare professionals
Ofoma	2021	Characteristics of U.S. Acute Care hospitals That Have implemented telemedicine Critical Care	no information on healthcare professionals, more hospital characteristics
Rakers	2023	Perceived barriers and facilitators of structural reimbursement for remote patient monitoring, an exploratory qualitative study	no information on factors related to healthcare professionals, but more to reimbursement problems
Ruxwana	2014	A Generic Quality Assurance model (GQAM) for successful e-health implementation in rural hospitals in South Africa	no reporting on effects/factors regarding healthcare professionals
Slevin	2019	A qualitative study of chronic obstructive pulmonary disease patient perceptions of the barriers and facilitators to adopting digital health technology	no information on healthcare professionals, aimed at patient experiences
Song	2021	A Comprehensive 6A framework for Improving Patient Self-Management of Hypertension Using mHealth Services: Qualitative Thematic Analysis	no information on healthcare professionals, aimed at patient perspectives
Steinberg	2022	Optimizing Engagement in an mHealth Intervention for Diabetes Support During Pregnancy: the Role of Baseline Patient Health and Behavioral characteristics	no information on healthcare professionals
Tieu	2015	Barriers and Facilitators to Online Portal Use Among Patients and Caregivers in a Safety Net Health Care System: A Qualitative Study	factors mentioned by healthcare professionals are related to patient use
Tseng	2018	Catalyzing healthcare transformation with digital health: Performance indicators and lessons learned from a digital Health Innovation Group	no information on healthcare professionals
Weinstein	2014	Telemedicine, telehealth, and mobile health applications that work: opportunities and barriers	unclear how information regarding healthcare professionals is gathered
Yang	2022	Artificial intelligence healthcare service resources adoption by medical institutions based on TOE framework	no information on healthcare professionals
Yu	2022	Design of an Integrated Acceptance framework for Older Users and eHealth: Influential Factor Analysis	no information on healthcare professionals
Zachrison	2020	Understanding barriers to telemedicine implementation in Rural Emergency Departments	no reporting on effects/factors regarding healthcare professionals, more to centre characteristics
Zailani	2014	Determinants of telemedicine acceptance in selected public hospitals in Malaysia: clinical perspective	no reporting on effects/factors regarding healthcare professionals, more to process

THEMES FOUND IN RE-ANALYSIS OF SCOPING REVIEW – TABLES S3A AND S3B, FIGURE S2

Table S3a. *Themes and subthemes mapped on the domains of the (modified) Work-System Model, and linked to the Quintuple Aim.*

ID	Domain*	Short theme†	Subtheme†	Quintuple Aim
A	Digital Health Technology	Value and Necessity	-	Enhancing the care experience
		Functionality	-	Enhancing the care experience
		Performance	-	Reducing costs
		Design and Scalability	-	Enhancing the care experience
		Supplementary Role and Knowledge	-	Enhancing the care experience
		Usability and User Experience	Acceptability and Flexibility	Enhancing the care experience
			Ease of Use and Understanding	Enhancing the care experience
			Usability	Enhancing the care experience
B	Organisation	Organisational Support and Leadership	Leadership and Champions	Care team well-being
			Stakeholder Involvement	Care team well-being
		Planning and Implementation	Strategic Planning	Advancing health equity
			Operational Strategies	Advancing health equity
		Resources	Workforce and Staffing	Reducing costs
			Financial and Material Resources	Reducing costs
C	Patient	Literacy	-	Improving population health
		Patient and Family factors	-	Enhancing the care experience
		Patient Outcomes	-	Enhancing the care experience
D	Healthcare Professional	Acceptance and Use	-	Care team well-being
		Traits	Resistance	Care team well-being
			Openness to change	Care team well-being
			Opinions	Care team well-being
			Engagement	Care team well-being
		Benefits	-	Care team well-being
		Colleagues and Communication	Negative Interaction	Care team well-being
			Positive Interaction	Care team well-being
			Leadership	Care team well-being
			Buy-in and peer influence	Care team well-being
		Competency	-	Care team well-being
		Experiences	Positive	Care team well-being

			experiences	
			Negative experiences	Care team well-being
			Ambivalent experiences	Care team well-being
		Fears, Beliefs and Concerns	About patients	Care team well-being
			Beliefs and positive thoughts	Care team well-being
			Fear and anxiety	Care team well-being
			Privacy, security and safety	Care team well-being
			Work and workload	Care team well-being
		Health	-	Care team well-being
		Perceptions	Awareness	Care team well-being
			Burden	Care team well-being
			Ease of Use	Care team well-being
			Gain	Care team well-being
			Preferences	Care team well-being
			Usefulness	Care team well-being
		Training, education and support	General	Care team well-being
			Skills	Care team well-being
E	Tasks	Operational Impact	-	Reducing costs
		Time Management	-	Reducing costs
		Workflow	-	Reducing costs
		Workload	Increased Workload	Care team well-being
			Decreased and Normalized Workload	Care team well-being
F	Environment	Equity and Sustainability	-	Advancing health equity
		Regulations	-	Enhancing the care experience
		Physical Environment	-	Enhancing the care experience

**Domain in (modified) Work System Model; †(Sub)theme from thematic analyses; HCP: Healthcare Professional; DHT: Digital Health Technologies*

Table S3b. *Themes and subthemes mapped on the interactions of the (modified) Work-System Model, and linked to the Quintuple Aim.*

ID	Interaction*	Short theme†	Subtheme†	Quintuple Aim
A-B	Digital Health Technology – Organisation	Data Security	-	Enhancing the care experience
		Support	Technical Support	Enhancing the care experience
			Organisational Support	Enhancing the care experience
A-C	Digital Health Technology – Patient	Access	-	Enhancing the care experience
		Suitability	-	Advancing health equity
A-D	Digital Health Technology – Healthcare Professional	Attitudes	Attitudes towards DHT	Care team well-being
			User readiness	Care team well-being
		Implementation and Operations	-	Enhancing the care experience
A-E	Digital Health Technology – Tasks	Comparing	-	Reducing costs
		Integration and Interoperability	-	Reducing costs
A-F	Digital Health Technology – Environment	Challenges in adopting	-	Advancing health equity
B-D	Organisation – Healthcare Professional	Interprofessional Collaboration	Collaboration and Team Dynamics	Care team well-being
			Communication	Care team well-being
			Facilitation of Interactions	Care team well-being
		Organisation and Operation	Involvement	Care team well-being
			Role and Scope	Care team well-being
			Senior help	Care team well-being
B-E	Organisation – Tasks	Identifying needs	-	Reducing costs
B-F	Organisation – Environment	Learning from and contacting other sites	-	Improving population health
C-D	Patient – Healthcare Professional	Access and consent	-	Enhancing the care experience
		Communication	-	Care team well-being
		Culture	-	Advancing health equity
		Information	-	Enhancing the care experience
		Patient-Provider Relationship	-	Care team well-being
		Quality	-	Enhancing the care experience
D-E	Healthcare Professional – Tasks	Workload Management	-	Care team well-being
		Workflow Adaptation	-	Care team well-being
D-F	Healthcare Professional – Environment	Guidelines and Regulations	-	Advancing health equity
		Financing and Collaborating	-	Advancing health equity
E-F	Tasks – Environment	n/a	n/a	n/a

**Interaction in (modified) Work System Model; †(Sub)theme from thematic analyses; HCP: Healthcare Professional; DHT: Digital Health Technologies*

762

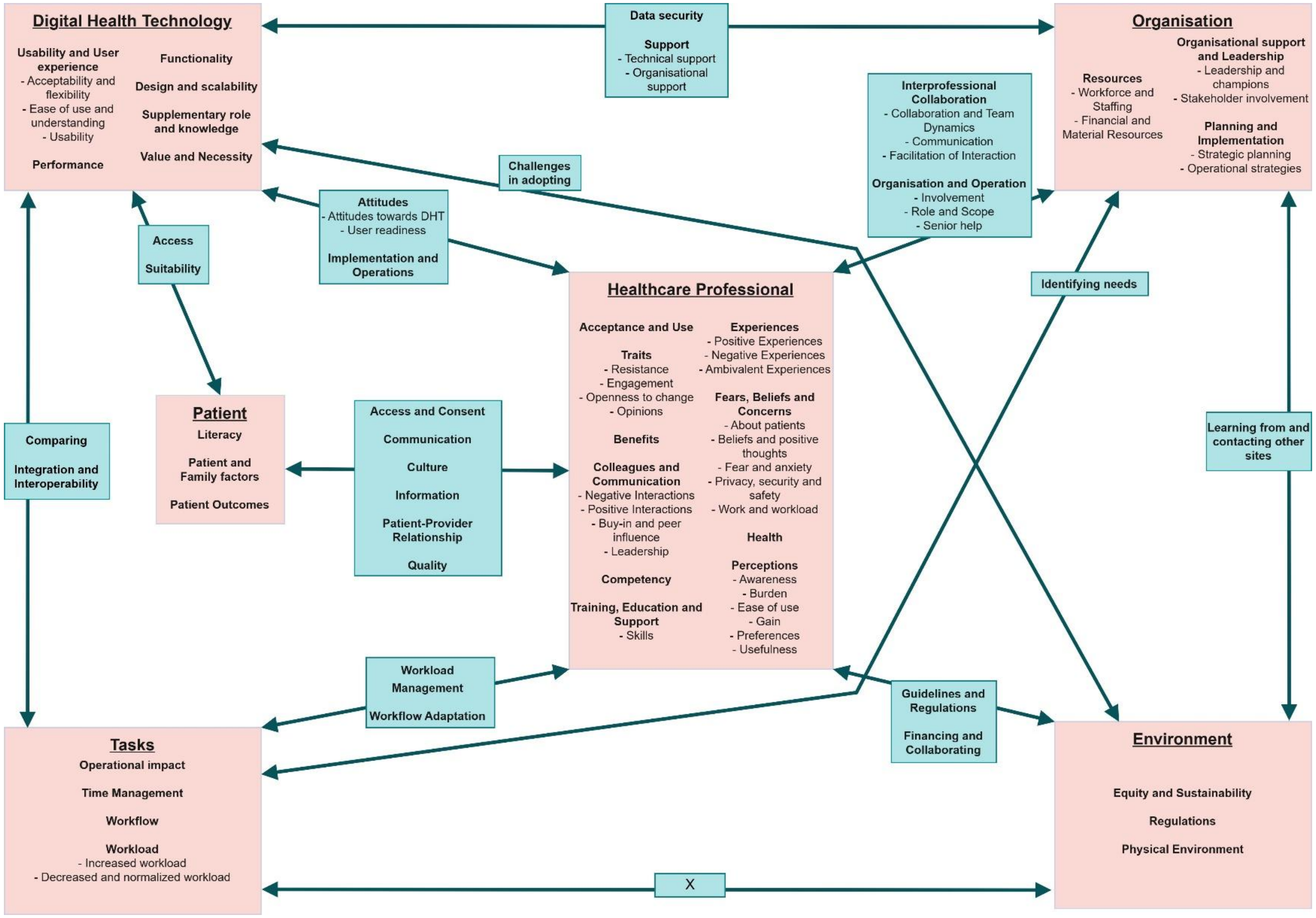

763

764 ***Figure S2****. Conceptual framework with themes. Schematically organised on a modified version of the Work-System Model. The pink boxes are domains in the*
765 *Work-System Model, on which themes and subthemes were mapped. Themes and subthemes regarding interactions between Work-System Model domains are*
766 *displayed in turquoise boxes mapped on the green arrows.*

RANKING OF THEMES ACCORDING TO THE REWEIGHTED PRIORITY-SETTING TOOL – TABLE S4

Table S4. *Full ranking, according to the REPS-tool of themes influencing DHT impact on health-care professionals.*

Rank	Theme
1	Perceived benefits of DHT use by health-care professionals
2	Acceptance and use of DHTs by health-care professionals
3	Involvement of health-care professionals in the implementation process
4	Presence of leaders and “champions”
5	Involvement of health-care professionals in the development process
6	Involvement of appropriate stakeholders
7	Training, education, and support
8	Training, education, and support: skills development
9	Communication between the organisation and health-care professionals
10	Clarity regarding the role of health-care professionals in the process
11	The patient–professional relationship
12	Leadership among healthcare professionals
13	Competence of the healthcare professional
14	The organization facilitating interactions
15	How communication between patient and professional unfolds
16	Support from “seniors” in the process
17	Changes in workflow due to DHT
18	Lower or normal workload with DHT
19	Coping with changed workload
20	User readiness
21	Provider perception: that DHT adds value
22	Provider perception: that DHT is easy to use
23	Fears, beliefs, and concerns: privacy, security, and safety
24	Positive interactions between healthcare professionals
25	Collaboration and team dynamics
26	Positive experiences of the healthcare professional with DHT
27	The healthcare professional’s attitude toward the technology
28	Healthcare professional personality traits: openness to change
29	Higher workload with DHT
30	Provider perception: the usefulness of DHT
31	Fears, beliefs, and concerns about patients, in relation to DHT use
32	Fears, beliefs, and concerns: the healthcare professional’s fear and uncertainty about DHT
33	Fears, beliefs, and concerns: positive thoughts and beliefs about DHT
34	Healthcare professional personality traits: work engagement
35	Healthcare professional personality traits: the professional’s opinion
36	Fears, beliefs, and concerns about work and workload
37	Health of the healthcare professional
38	Provider perception: that DHT is a burden
39	Provider perception: awareness of the presence of DHT
40	Healthcare professional personality traits: resistance
41	Provider perception: the provider’s preferences
42	Negative experiences of the healthcare professional with DHT
43	Ambivalent experiences of the healthcare professional with DHT
44	Negative interactions between healthcare professionals

REPS: Reweighted Priority-Setting; DHT: digital health technology

RANKING OF THEMES, UNWEIGHTED SUM SCORE, SENSITIVITY CHECK– TABLE S5

Table S5. *Full unweighted sum score ranking of themes influencing DHT impact on health-care professionals.*

Unweighted rank*	RRV-rank**	Theme	Sum score
1	1	Perceived benefits of DHT use by health-care professionals	52
2	4	Presence of leaders and “champions”	51
	2	Acceptance and use of DHTs by health-care professionals	51
	3	Involvement of health-care professionals in the implementation process	51
5	5	Involvement of health-care professionals in the development process	50
6	6	Involvement of appropriate stakeholders	49
7	7	Training, education, and support	47
8	8	Training, education, and support: skills development	46
9	9	Communication between the organisation and health-care professionals	45
10		Leadership among healthcare professionals	44
		Competence of the healthcare professional	44
		The organization facilitating interactions	44
	10	Clarity regarding the role of health-care professionals in the process	44
		The patient–professional relationship	44
15		Support from “seniors” in the process	43
		How communication between patient and professional unfolds	43
		Changes in workflow due to DHT	43
18		Lower or normal workload with DHT	42
		User readiness	42
		Coping with changed workload	42
21		Provider perception: that DHT adds value	41
22		Provider perception: that DHT is easy to use	40
23		Positive interactions between healthcare professionals	39
		Fears, beliefs, and concerns: privacy, security, and safety	39
		Collaboration and team dynamics	39
26		Positive experiences of the healthcare professional with DHT	38
27		The healthcare professional’s attitude toward the technology	37
28		Healthcare professional personality traits: openness to change	36
29		Provider perception: the usefulness of DHT	35
30		Fears, beliefs, and concerns about patients, in relation to DHT use	34
		Fears, beliefs, and concerns: positive thoughts and beliefs about DHT	34
		Fears, beliefs, and concerns: the healthcare professional’s fear and uncertainty about DHT	34
		Higher workload with DHT	34
34		Health of the healthcare professional	33
		Healthcare professional personality traits: work engagement	33
		Healthcare professional personality traits: the professional’s opinion	33
37		Fears, beliefs, and concerns about work and workload	32
		Provider perception: that DHT is a burden	32

39	Provider perception: awareness of the presence of DHT	31
40	Provider perception: the provider’s preferences	28
	Healthcare professional personality traits: resistance	28
42	Negative experiences of the healthcare professional with DHT	25
	Ambivalent experiences of the healthcare professional with DHT	25
44	Negative interactions between healthcare professionals	23

**: Based on the sum score from all participants on each item*

***: Based on reweighted range voting. Only the top-10 ranked items are displayed.*

DHT: digital health technology
