# Supplementary figures and images for: Towards responsible digital health implementation: A mixed-methods exploratory study developing a tool to assess workforce experience
Source: Digit Health. 2026 Jul 6;12:20552076261450419. doi: 10.1177/20552076261450419 (PMC13338532; doi:10.1177/20552076261450419)

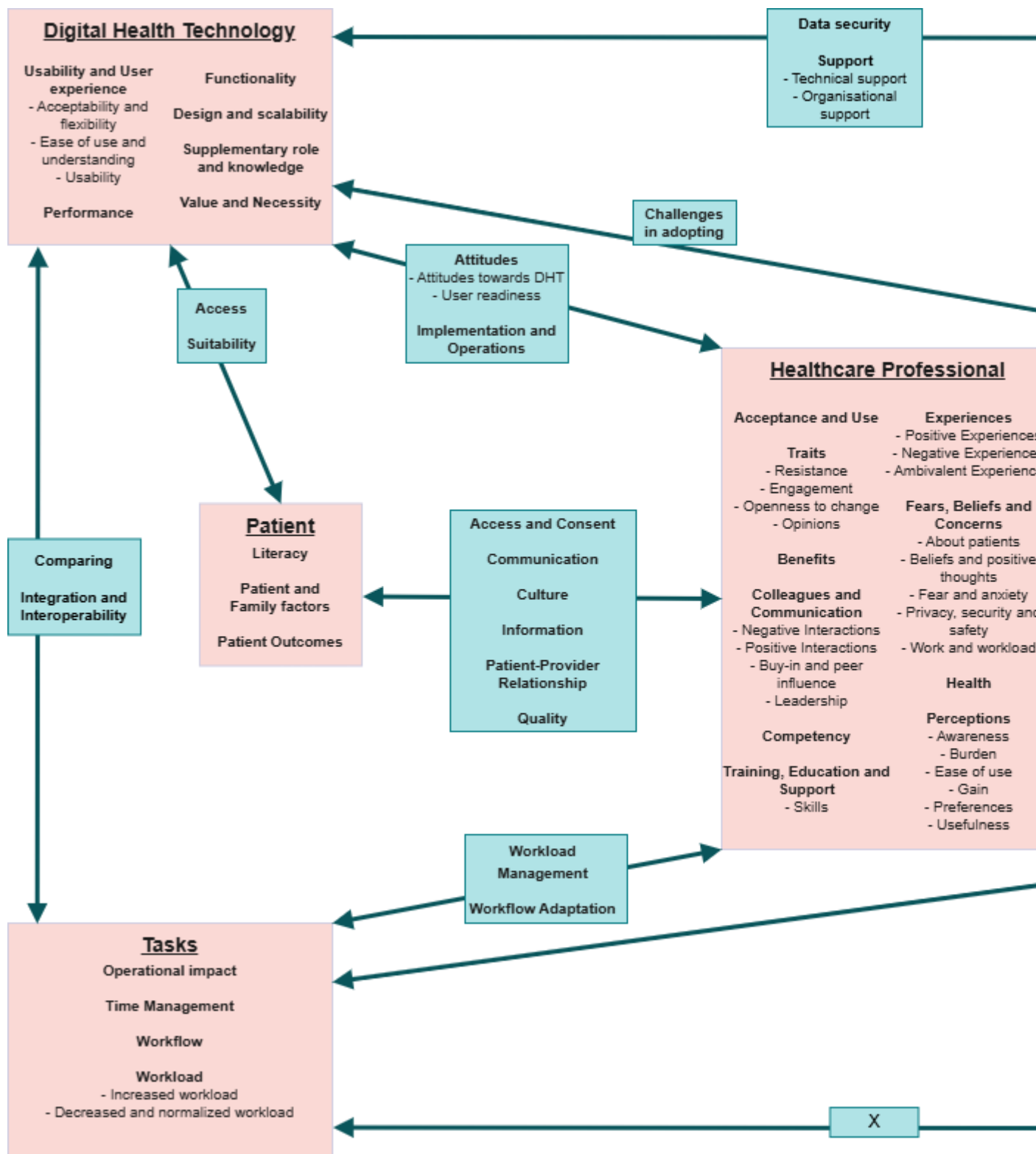

Supplement: Supplemental material - Towards responsible digital health implementation: A mixed-methods exploratory study developing a tool to assess workforce experience [file sj-pdf-2-dhj-10.1177_20552076261450419.pdf]
